# Parkinson’s disease patients have a complex phenotypic and functional Th1 bias: cross-sectional studies of CD4+ Th1/Th2/T17 and Treg in drug-naïve and drug-treated patients

**DOI:** 10.1186/s12974-018-1248-8

**Published:** 2018-07-12

**Authors:** Natasa Kustrimovic, Cristoforo Comi, Luca Magistrelli, Emanuela Rasini, Massimiliano Legnaro, Raffaella Bombelli, Iva Aleksic, Fabio Blandini, Brigida Minafra, Giulio Riboldazzi, Andrea Sturchio, Marco Mauri, Giorgio Bono, Franca Marino, Marco Cosentino

**Affiliations:** 1 Center of Research in Medical Pharmacology, University of Insubria, Via Ottorino Rossi n. 9, 21100 Varese, VA Italy; 2 Movement Disorders Centre, Neurology Unit, Department of Translational Medicine, University of Piemonte Orientale, Novara, Italy; 3 Center for Research in Neurodegenerative Diseases, “C. Mondino” National Neurological Institute, Pavia, Italy; 4 Department of Biotechnology and Life Sciences, University of Insubria, Varese, Italy

**Keywords:** Parkinson’s disease, CD4+ T lymphocytes, Th1, Th2, Th17, Treg

## Abstract

**Background:**

Parkinson’s disease (PD) affects an estimated 7 to 10 million people worldwide, and only symptomatic treatments are presently available to relieve the consequences of brain dopaminergic neurons loss. Neuronal degeneration in PD is the consequence of neuroinflammation in turn influenced by peripheral adaptive immunity, with CD4+ T lymphocytes playing a key role. CD4+ T cells may however acquire proinflammatory phenotypes, such as T helper (Th) 1 and Th17, as well as anti-inflammatory phenotypes, such as Th2 and the T regulatory (Treg) one, and to what extent the different CD4+ T cell subsets are imbalanced and their functions dysregulated in PD remains largely an unresolved issue.

**Methods:**

We performed two cross-sectional studies in antiparkinson drug-treated and drug-naïve PD patients, and in age- and sex-matched healthy subjects. In the first one, we examined circulating Th1, Th2, Th17, and in the second one circulating Treg. Number and frequency of CD4+ T cell subsets in peripheral blood were assessed by flow cytometry and their functions were studied in ex vivo assays. In both studies, complete clinical assessment, blood count and lineage-specific transcription factors mRNA levels in CD4+ T cells were independently assessed and thereafter compared for their consistency.

**Results:**

PD patients have reduced circulating CD4+ T lymphocytes, due to reduced Th2, Th17, and Treg. Naïve CD4+ T cells from peripheral blood of PD patients preferentially differentiate towards the Th1 lineage. Production of interferon-γ and tumor necrosis factor-α by CD4+ T cells from PD patients is increased and maintained in the presence of homologous Treg. This Th1-biased immune signature occurs in both drug-naïve patients and in patients on dopaminergic drugs, suggesting that current antiparkinson drugs do not affect peripheral adaptive immunity.

**Conclusions:**

The complex phenotypic and functional profile of CD4+ T cell subsets in PD patients strengthen the evidence that peripheral adaptive immunity is involved in PD, and represents a target for the preclinical and clinical assessment of novel immunomodulating therapeutics.

**Electronic supplementary material:**

The online version of this article (10.1186/s12974-018-1248-8) contains supplementary material, which is available to authorized users.

## Background

Parkinson’s disease (PD) is among the most common neurological diseases, affecting 1–2 per 1000 in the general population with increasing prevalence with age, and up to 1 in 100 individuals above 60 years of age, resulting in an estimated 7 to 10 million people with PD worldwide [1, 2]. People with PD suffer from motor symptoms, including bradykinesia, rigidity, resting tremor, and postural instability, as well as from non-motor symptoms, such as autonomic disturbances, depression, and cognitive impairment, resulting in dramatically poor quality of life and rising economic burden for patients, caregivers, and the healthcare systems [3–5]. Available treatments for PD are only symptomatic and aim at relieving the loss of brain dopaminergic neurons by using the dopamine (DA) precursor L-DOPA, some dopaminergic agonists as well as other indirect dopaminergic agents [6]. Dopaminergic replacement improves patients’ quality of life, although as disease progresses non-motor and motor symptoms emerge that may be resistant to dopaminergic medications [7, 8]. No treatments currently exist to prevent PD or delay its progression, mainly due to the still limited comprehension of the events ultimately leading to neurodegeneration.

PD is characterized by the progressive loss of dopaminergic neurons in the *substantia nigra pars compacta* and by the appearance of Lewy bodies, which are intracellular inclusions of aggregated α-synuclein [9–12]. Despite extensive knowledge about the mechanisms leading to neuronal death, which include mitochondrial dysfunction, oxidative, and proteolytic stress, and neuroinflammation, understanding the causes of neurodegeneration in PD remains so far an elusive goal. In this regard, novel clues are possibly coming from evidence concerning the role of peripheral adaptive immunity in the regulation of neuroinflammation [13–16]. T cells indeed can be found in the *substantia nigra* of parkinsonian brains [17, 18]. Both CD8+ and CD4+ T cells (but not B cells) occur in postmortem brain specimens from PD patients as well as in the 1-methyl-4-phenyl-1,2,3,6-tetrahydropyridine (MPTP) mouse model of PD, and evidence from the mouse model indicates that CD4+ T cells determine T cell-mediated dopaminergic cell death [18]. Although T lymphocytes infiltrate parkinsonian brains, decreased numbers of CD3+ and CD4+ T lymphocytes have been consistently reported in peripheral blood of PD patients [19].

CD4+ T lymphocytes play a crucial role in the orchestration of an effective immune response during host defense as well as in the pathogenesis of inflammatory disease. To this end, CD4+ T cells may acquire proinflammatory phenotypes, such as T helper (Th) 1 and Th17, as well as anti-inflammatory phenotypes, such as Th2 and the T regulatory (Treg) 1 [20, 21], and evidence from animal models of PD suggests that Th1 and Th17 may be detrimental while Th2 and Treg may be protective (reviewed in [22]). Whether CD4+ T cell subsets are imbalanced and their functions are dysregulated in PD patients remains however largely an unresolved issue.

The aim of this study was to investigate the profile of Th1, Th2, Th17, and Treg in the peripheral blood of PD patients, either drug-naïve or on dopaminergic replacement therapy, and to examine their function. Since we previously found that in PD patients CD4+ T naïve cells D_1_-like dopaminergic receptors (DR) decrease, while in T memory cells D_2_-like DR increase, with increasing motor disability [23], we also assayed DR on all CD4+ T cells subsets. To this end, we conducted two clinical studies, recruiting PD patients and age- and sex-matched healthy subjects. In the first study, Th1, Th2, and Th17 count and function were examined, while in the second study, Treg frequency and function were assessed. In both protocols, complete clinical assessment, blood count, and lineage-specific transcription factors mRNA levels in CD4+ T cells were independently assessed and thereafter compared for their consistency. Results provide for the first time a detailed phenotypic and functional profile of the CD4+ T cell compartment in peripheral blood in both drug-naïve PD patients and in patients on dopaminergic drugs, which extends available knowledge about the involvement of CD4+ T cell subsets in PD and offers an array of biomarkers for the assessment of novel unconventional therapeutics targeting peripheral immunity.

## Methods

### Subjects

Peripheral venous blood samples were collected from patients with idiopathic PD [24], either drug-naïve (PD-dn, i.e., PD patients who never received any antiparkinson drugs) or on antiparkinson drug treatment (PD-dt), and from age- and sex-matched healthy subjects (HS). PD was diagnosed according to the United Kingdom Parkinson’s Disease Society Brain Bank Criteria. Exclusion criteria were history of autoimmune or inflammatory disorders and chronic immunosuppressive treatment.

Participants were recruited at the Movement Disorders Center of the University of Piemonte Orientale, Divisione di Neurologia, Ospedale Maggiore of Novara, at the Interdepartmental Research Center for Parkinson’s Disease of the Neurological Institute “C. Mondino” of Pavia, and at the Centre for Parkinson’s Disease and Movement Disorders of the Neurological Service at the Ospedale di Circolo of Varese, Italy. HS were spouses and caregivers of enrolled PD patients.

The Ethics Committees of Ospedale di Circolo of Varese and Neurological Institute “C. Mondino” of Pavia approved the protocol and all the participants signed a written informed consent before enrollment. The study was performed according to the Declaration of Helsinki and to the relevant ethical guidelines for research on humans.

After enrollment, subjects were submitted to a complete examination. PD patients were staged according to the criteria of Hoehn and Yahr (H&Y) [25] and evaluated by means of the Unified Parkinson’s Disease Rating Scale (UPDRS) part III [26]. Antiparkinson drug doses were recorded at the time of enrollment and l-DOPA equivalent doses (LED) were calculated according to established guidelines [27].

Withdrawal of venous blood was performed after a fasting night, between 8:00 a.m. and 10:00 a.m., in EDTA-coated tubes (BD Vacutainer). Tubes were subsequently coded and stored at room temperature until processing, which occurred 24 h after collection, to ensure homogeneous treatment of all the samples. Complete blood cell count with differential analysis was conducted on separate blood samples collected in EDTA-coated tubes (BD Vacutainer).

### Reagents

Bovine serum albumin (BSA) and 4-(2-hydroxyethyl)-1-piperazineethanesulfonic acid (HEPES) were purchased from Sigma, Italy. RPMI 1640, heat-inactivated fetal bovine serum (FBS), glutamine, and penicillin/streptomycin were obtained from Euroclone, Italy. Ficoll-Paque Plus was from Pharmacia Biotech (Uppsala, Sweden, code GEH1714403). Purified mouse ab anti-human CD3 (code 555330, clone UCHT1, Mouse IgG1, κ) and purified mouse ab anti-human CD28 (code 555726, clone CD28.2, Mouse IgG1, κ) were obtained from Becton Dickinson, Italy. Phytohaemagglutinin (PHA, code L8902), recombinant interleukin (IL)-2 (code 0208AF12), dopamine hydrochloride (code H8502), phorbol 12-myristate 13-acetate (PMA, code P813), and ionomycin (code I3909) were all from Sigma-Aldrich (Saint Louis, MO, USA).

Human ab anti-INF-γ (code 130-095-743) and anti-IL-4 (code 130-095-753), IL-1β (code 130-093-895), IL-4 (code 130-095-373), IL-6 (code 130-095-365), IL-12 (code 130-096-704), and TGF-β (code 130-095-067) were from Miltenyi Biotec, Bergisch Gladbach, Germany.

ELISA kits for human interferon (IFN)-γ (code EHIFNG), tumor necrosis factor (TNF)-α (code EH3THFA), IL-4 (code EHIL4), IL-10 (code EHIL10), and IL-17A (code EHIL17A) were all from Thermo Scientific, Rockford, USA.

### Flow cytometric analysis of CD4+ T helper cells in whole blood

Analysis was performed in two steps. In the first step, 100 μl aliquots of whole blood were prepared from each subject (five were used for DR staining, one as control for the secondary PE-goat anti-rabbit (PEGAR) ab, and 1 as negative control, i.e., without any ab). All the aliquots were incubated with a cocktail of anti-human CD4, CXCR3 (CD183), CCR4 (CD194), and CCR6 (CD196) ab for the identification of CD4+ T lymphocytes and of the following CD4+ T helper subsets: Th1 cells (CD4 + CXCR3 + CCR4-CCR6-), Th2 cells (CD4 + CXCR3-CCR4 + CCR6-), Th17 cells (CD4 + CXCR3-CCR4 + CCR6+), and Th1/17 cells (CD4 + CXCR3 + CCR4-CCR6+). After 20 min in the dark, erythrocytes were removed by means of a lysis buffer ((g/L) NH_4_Cl 8.248, KHCO_3_ 1.0, EDTA 0.0368). Samples were then centrifuged, supernatants were removed, and cells were washed in PBS ((g/L) NaCl 8.0, KCl 0.2, Na_2_HPO_4_ 1.42, KH_2_PO_4_ 0.24, pH 7.4) supplemented with 1% BSA (PBS/BSA) and resuspended in PBS/BSA. Total leukocytes were counted by means of a hemocytometer and cell viability, determined by the Trypan blue exclusion test, was always > 99%. During the second step, each aliquot was stained for one of the five DR by an indirect labeling procedure. Briefly, samples were stained with primary ab and incubated for 30 min on ice. After washing, samples were incubated with PEGAR ab for 30 min on ice in the dark. Samples were then washed and resuspended in 350 μl PBS and left on ice until acquisition. Ab used in the study are listed in Additional file 1: Table S1, and the gating strategy is shown in Additional file 2: Figure S1. Acquisition was then performed on a BD FACSCanto II flow cytometer (Becton Dickinson, Milan, Italy) with BD FACSDiva software (version 6.1.3). Lymphocytes were identified by means of their classical forward scatter (FSC) and side scatter (SSC) signals, and a minimum of 20,000 lymphocytes from each sample was collected in the gate. Data were analyzed with the FlowJo software (version 8.3.2). The results were finally expressed as absolute numbers (10^6^/mL) as well as percentage of positive cells (%).

### Flow cytometric analysis of CD4+ Treg subsets in whole blood

Analysis of CD4+ Treg was performed according to Miyara et al. [28]. Briefly, 100 μl aliquots of whole blood were prepared as above described, and 7 aliquots of 100 μL were prepared from each subject (five for DR staining, one as control for the secondary PEGAR ab, and one as negative control). The staining protocol consisted of two steps. During the first step, each aliquot was stained for one of the five DR by an indirect labeling procedure (primary ab + secondary ab labeled with PE). During the second step, all the aliquots were incubated with a cocktail of anti-human CD4, CD25, CD127, and CD45RA ab for the identification of CD4+ T lymphocytes, of total Treg (cTreg, CD4 + CD25^high^CD127^low^), and of naïve Treg (nTreg, CD4 + CD25^high^CD127^low^CD45RA+) and activated Treg (aTreg, CD4 + CD25^high^CD127^low^CD45RA-). Ab used in the study are listed in Additional file 1: Table S1, and the gating strategy is shown in Additional file 2: Figure S2. Acquisition, lymphocyte identification, data analysis, and final expression of results were performed as described above.

### Isolation of peripheral blood mononuclear cells (PBMC)

PBMC were isolated from whole blood by using Ficoll-Paque Plus density gradient centrifugation. Cells were resuspended and, if necessary, any residual contaminating erythrocytes were lysed by addition of 5 mL of lysis buffer ((g/L) NH_4_Cl 8.248, KHCO_3_ 1.0, EDTA 0.0368) followed by incubation for 5 min, during which samples were gently vortexed, and centrifugation at 100 g for 10 min at room temperature (RT). Cells were washed twice in PBS by addition of 15 mL of PBS and centrifugation at 300 g and 10 min at RT, and resuspended at the final concentration of 1 × 10^6^ cells/mL of RPMI/10% FBS for subsequent culture. Typical PBMC preparations contained at least 80% lymphocytes, as assessed by flow cytometry. Cell viability, assessed by the trypan blue exclusion test was always > 99%.

### Real-time PCR assays of CD4+ T cells

CD4+ T cells were isolated from PBMC by means of *Dynabeads CD4 Positive Isolation kit* (Life Technologies, code 11145D). At least 50,000 CD4+ T cells were thereafter resuspended in PerfectPure RNA lysis buffer (5 Prime GmbH, Hamburg, Germany), and total RNA was extracted by PerfectPure RNA Cell Kit™ (5 Prime GmbH, code 2302340). The amount of extracted RNA was estimated by spectrophotometry at λ = 260 nm. Total mRNA was then reverse-transcribed using a random primer and a high-capacity cDNA RT kit (Applied Biosystems, code 4368813), and the resulting amount of cDNA was estimated by spectrophotometry at λ = 260 nm. Real-Time PCR reactions were then started with 1 μM cDNA. Amplification of cDNA was performed by means of the SsoAdvanced™ Universal Probes Supermix (BIORAD, code 1725282) for the analysis of mRNA levels of the transcription factor genes *TBX21*, *STAT1*, *STAT3*, *STAT4*, *STAT6*, *RORC*, *GATA3*, *FOXP3*, and *NR4A2*. cDNA was assayed on StepOne® System (Applied Biosystems). Real-time PCR conditions are shown in Additional file 3: Table S2.

Linearity of assays was tested by constructing standard curves using serial 10-fold dilutions of a standard calibrator cDNA for each gene. Regression coefficients (*r*^2^) were always > 0.999. Assays were performed in triplicate for each sample, and levels of mRNA were finally expressed as 2^−ΔCt^ where ΔCt = [Ct (sample)−Ct (housekeeping gene)]. Relative expression was determined by normalization to expression of *RPS18*, which is the gene for 18S cDNA. Data analysis was performed by StepOne software™ 2.2.2 (Applied Biosystems).

### Separation of naïve CD4+ T cells and polarization assay

The isolation of naïve CD4+ T cells from PBMC was performed using the human CD4+ Naïve T cell Isolation Kit (Miltenyi Biotec, code 130-094-131), and polarization was assayed by the Human Th1/Th2/Th17 Phenotyping Kit (BD, code 560751), according to the manufacturers’ instructions. Purity of separated naïve CD4+ T lymphocytes was always more than 95%, as confirmed by flow cytometric analysis.

The polarization assay was developed according to published methods [29–31] with modifications. Briefly, naïve CD4+ T cells were cultured in U-bottomed 96-well plates primed with anti-CD3/CD28 ab, in standard conditions (Th0), or under the different following polarizing conditions: IL-12 (10 ng/mL) and anti–IL-4 ab (10 μg/mL) for Th1; IL-4 (10 ng/mL) and anti-IFN-γ ab (10 μg/mL) for Th2; IL-1β (10 ng/mL), IL-6 (50 ng/mL), TGF-β (5 ng/mL), anti–IL-4 ab (10 μg/mL), and anti-IFN-γ ab (10 μg/mL) for Th17. Cells were incubated for 4 days at 37 °C in a moist atmosphere of 5% CO_2_. Cells were thereafter observed under light microscopy to confirm formation of clusters indicating cell activation. Wells were then replenished with 500 μL fresh medium containing human recombinant IL-2 (10 ng/mL for Th1 and Th2, and 2 ng/mL for Th17), and left in the incubator for another 3 days. Cells were subsequently collected, centrifuged at 600×*g* for 5 min, resuspended in 1 mL RPMI with 10% FBS, adjusted to the final concentration of 1 × 10^6^ cells/mL for each sample, and stimulated with PMA (50 ng/mL), ionomycin (1 μg/mL), and GolgiStop™ Protein Transport Inhibitor (included in the Phenotyping Kit) for 5 h at 37 °C in 5% CO_2_. Cells were finally harvested, stained with the Human Th1/Th2/Th17 Phenotyping Kit, and analyzed by flow cytometry according to the manufacturer’s protocol.

### Purification and functional assays of CD4+ T regulatory (Treg) and T effector (Teff) cells

Treg and Teff were purified from PBMC by means of the human CD4 + CD25+ Regulatory T cell Isolation Kit (Miltenyi Biotec, code 130-091-301), according to the manufacturer’s instructions. Both Treg and Teff viability were more than 99% as assessed by the trypan blue exclusion test. Flow cytometric analysis showed that CD4 + CD25^high^CD127^low^ Treg cells were 7.1 ± 0.2% in the Teff fraction and 76.1 ± 3.2% in the Treg fraction (mean±SD, *n* = 3). Treg and Teff cells were placed in U-bottomed 96-well plates at the concentration of 1 × 10^6^/mL in RPMI 1640 medium supplemented with 10% heat-inactivated FBS, 2 mM glutamine, and 100 U/mL penicillin/streptomycin at 37 °C in a moist atmosphere of 5% CO_2_. Treg and Teff were cultured alone or in co-culture in different Teff:Treg ratios (1:1, 1:0.5, 1:0.25, and 1:0.125) in resting conditions or added with PHA (5 μg/mL) and IL-2 (40 ng/mL). Measurement of cell proliferation was performed after 5 days, by means of standard staining with Cell Proliferation Dye eFluor 670 (eBioscience-Prodotti Gianni, Italy, code 65-0840) and flow cytometric analysis. Production of IFN-γ, TNF-α, IL-4, IL17A, IL-10, and TGF-β was assessed in supernatants, which were collected after 48 h and frozen at − 80 °C until analysis, performed by means of standard ELISA assays.

### Statistical analysis

Distribution of the values was assessed by the D’Agostino and Pearson normality test. Statistical significance of the differences between HS and PD patients and between PD-dn and PD-dt patients was then analyzed by means of two-tailed Student’s *t* test or by the Mann-Whitney test, as appropriate, for continuous variables, and by the Fisher’s exact test for categorical variables. Correlations among continuous variables were assessed by Pearson or Spearman correlation analysis. Differences between HS and PD patients categorized for UPDRS part III score or H&Y stage were analyzed by ordinary one-way ANOVA or by the Kruskal-Wallis test, with either Holm-Sidak’s or Dunn’s adjustments for multiple comparisons, and trend analysis in PD patients was performed by ANOVA post test for linear trend. Calculations were performed using commercial software (GraphPad Prism version 5.00 for Windows, GraphPad Software, San Diego, CA, USA, www.graphpad.com).

## Results

### Study #1-CD4+ T cells and Th subsets in HS and PD patients

#### Subjects

The study included 47 HS and 82 PD patients (Table 1). Patients comprised 26 subjects who had never been treated with antiparkinson drugs before enrollment (drug-naïve PD patients, PD-dn), and 56 patients on antiparkinson drugs (drug-treated PD patients, PD-dt). In comparison to PD-dn patients, a larger fraction of PD-dt patients had UPDRS part III score higher than 10 and H&Y stage 1.5 or more. Disease duration of PD-dt patients was 5.5±4.7 years (range 0.5–22 years). Complete blood counts of HS and PD patients were all within normal limits (Additional file 4: Table S3A); however, in comparison to HS, both PD-dn and PD-dt patients had less total lymphocytes (on average, about − 19% in PD-dn and − 16% in PD-dt patients). Complete blood count did not differ between PD-dn and PD-dt patients (Additional file 4: Table S3A).

**Table 1:** Characteristics of HS and PD patients enrolled in study #1. Data are means ± SD unless otherwise indicated

	HS	PD-dn	PD-dt	P		
				HS vs PD-dn	HS vs PD-dt	PD-dn vs PD-dt
n47		26	56			
Gender (M/F)	25/22	15/11	34/22	0.808	0.549	0.813
Age (years)	66.8 ± 10.3	68.0 ± 9.3	70.4 ± 8.5	0.632	0.057	0.254
UPDRS part III (score)		13.6 ± 7.2	15.3 ± 6.6			0.101
1–10 (*n*)		14	13			0.010
11–20 (*n*)		7	34			
> 20 (*n*)		5	9			
H&Y scale (stage)		1.3 ± 0.5	1.9 ± 0.7			0.001
1.0 (*n*)		16	14			0.004
1.5–2.0 (*n*)		9	31			
> 2.5 (*n*)		1	11			
LED (mg/day)			533.2 ± 360.1			
Drugs						
l-DOPA (*n*)			45^a^			
DA agonists (*n*)			36^b^			
Pramipexole (*n*)			23			
Ropinirole (*n*)			5			
Rotigotine (*n*)			8			
Rasagiline (*n*)			21			

^a^11 taking l-DOPA alone, and 34 taking l-DOPA + DA agents

^b^9 taking DA agonists alone (4) or with rasagiline (5), and 34 taking DA agonists + l-DOPA, without (19) or with rasagiline (15)

#### Circulating CD4+ T cells and Th subsets

**Fig. 1:**
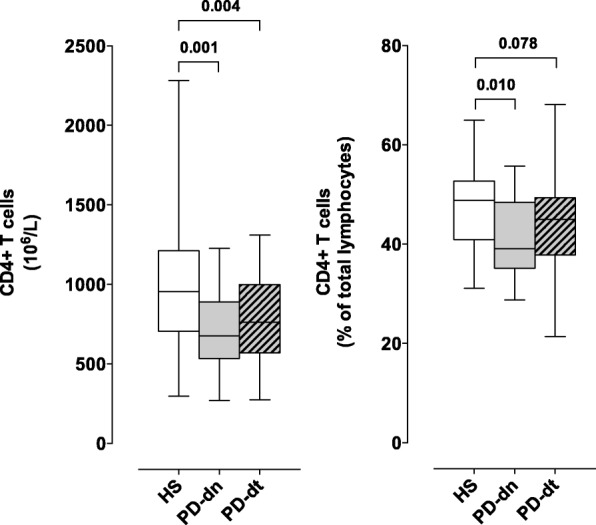
CD4+ T cells in HS and PD patients. Cells are shown as absolute numbers (left panel) and as percentage of total lymphocytes (right panel). Data are medians with 25°–75° percentiles (boxes) and min–max values (whiskers)

**Fig. 2:**
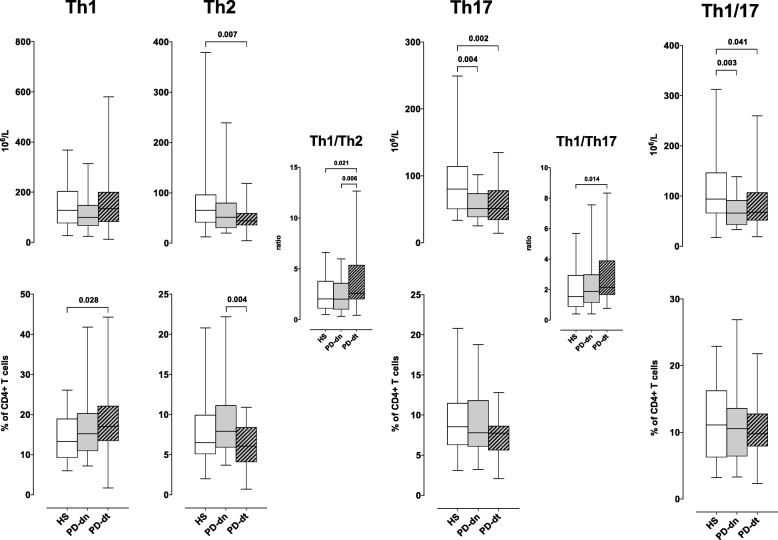
Th subsets in HS and PD patients. Cells are shown as absolute numbers (upper panels) and as percentage of total lymphocytes (lower panels). Th1/Th2 ratio and Th1/Th17 ratio are also shown (middle panels). Data are medians with 25°–75° percentiles (boxes) and min–max values (whiskers)

In both PD-dn and PD-dt patients, CD4+ T cells were less than in HS, both as absolute counts and as percentage of total lymphocytes. There was on the contrary no difference between PD-dn and PD-dt patients (Fig. 1). Th1 cells did not differ between HS and PD patients in terms of absolute counts, but their frequency among CD4+ T cells was higher in PD patients (18.1 ± 9.0% vs 14.6 ± 6.1%, *P* < 0.042), mainly due to increased proportion in PD-dt patients (Fig. 2). Th2 cells were less in PD patients in comparison to HS (54.9 ± 35.2 × 10^6^/L vs 78.7 ± 63.7 × 10^6^/L, *P* < 0.017), due to decreased count in PD-dt patients, and their frequency was lower in PD-dt in comparison to PD-dn patients (Fig. 2). Absolute counts (but not frequencies) of Th17 and Th1/17 cells were less in both PD-dn and PD-dt patients in comparison to HS (Fig. 2). In comparison to HS, PD patients as a whole showed similar Th1/Th2 ratio (2.94 ± 2.15 vs 2.48 ± 1.70 in HS, *P* = 0.292) but higher Th1/Th17 ratio (2.69 ± 1.85 vs 2.00 ± 1.40 in HS, *P* = 0.040). In comparison to PD-dn patients, PD-dt patients had higher Th1/Th2 ratio and similar Th1/Th17 ratio (Fig. 2). Finally, there were no major differences in DR expression on any Th subsets among HS, PD-dn, and PD-dt patients (Additional file 2: Figures S3-S6).

#### Transcription factors mRNA levels in CD4+ T cells

**Fig. 3:**
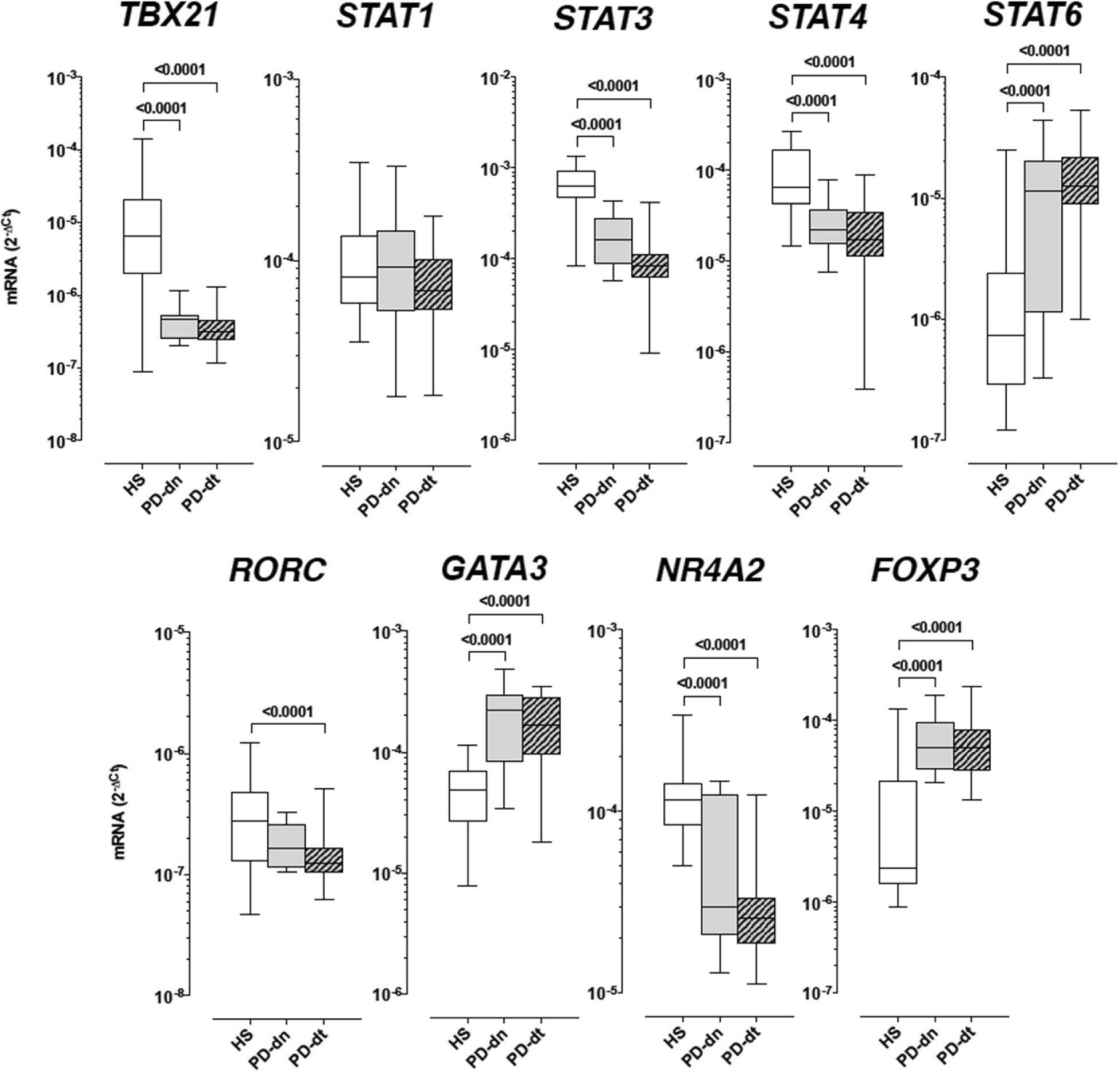
Transcription factors mRNA levels in CD4+ T cells of HS and PD patients. Data are medians with 25°–75° percentiles (boxes) and min–max values (whiskers)

In comparison to HS, both PD-dn and PD-dt patients had lower levels of *TBX21*, *STAT3*, *STAT4*, and *NR4A2*, and higher levels of *STAT6*, *GATA3*, and *FOXP3* (Fig. 3). *RORC* was also lower in PD-dn and PD-dt patients in comparison to HS; however, the difference reached the statistical significance in PD-dt patients only. There was no difference in *STAT1* expression among HS, PD-dn, or PD-dt patients (Fig. 3).

#### Polarization of naïve CD4+ T cells

**Fig. 4:**
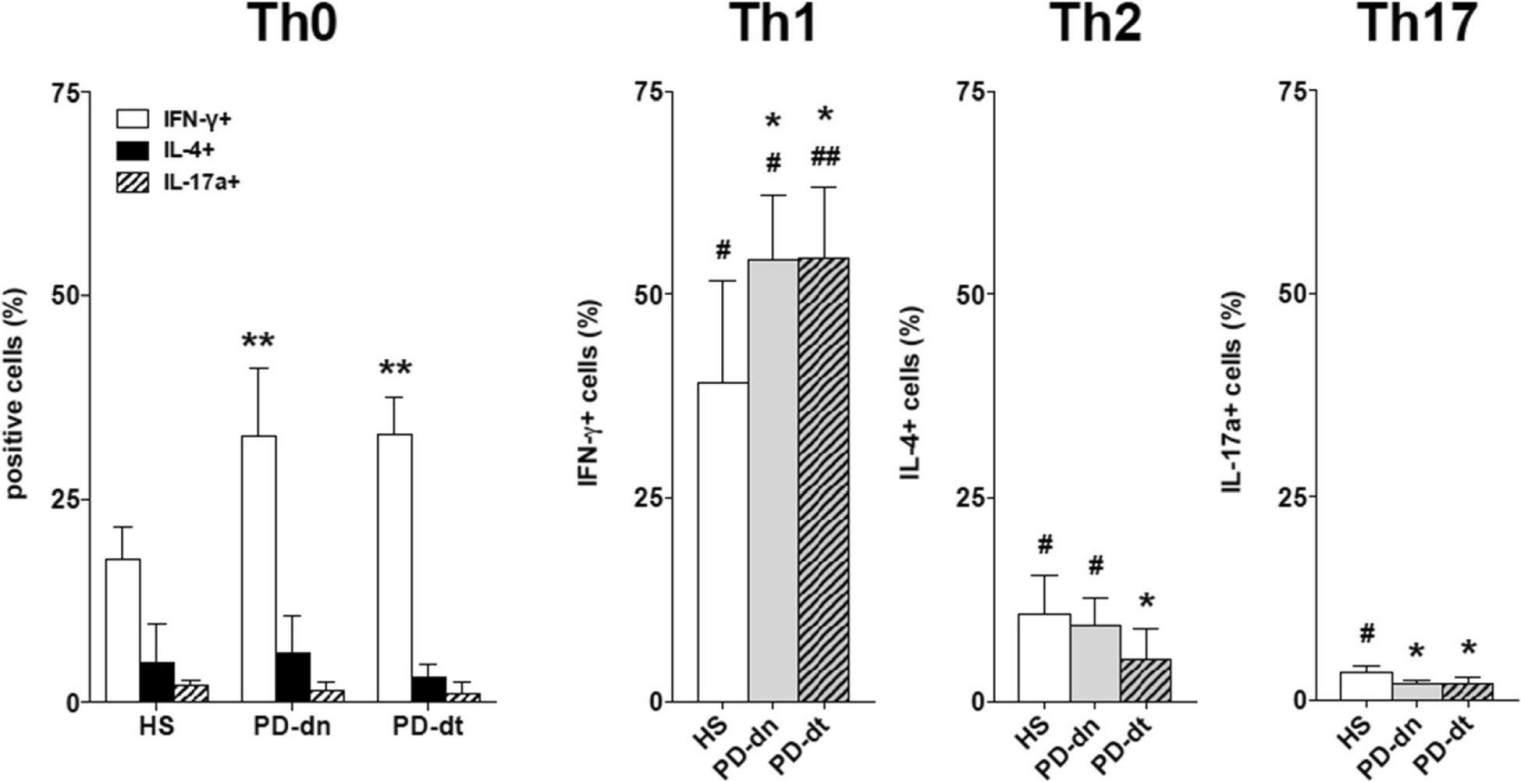
Polarization of naïve CD4+ T cells towards Th1, Th2, and Th17 in HS and PD patients. Data are means ± SD of *n* = 5–6 subjects tested in triplicate. * = *P* < 0.05 and ** = *P* < 0.01 vs HS; # = *P* < 0.05 and ## = *P* < 0.01 vs standard conditions (Th0)

Under standard conditions, the proportion of IFN-γ-positive Th0 cells was nearly two-fold in PD patients in comparison to HS (on average, + 85% in PD-dn and + 87% in PD-dt patients), while there was no difference in either IL-4- or IL17A-positive cells (Fig. 4). Th1 polarizing conditions resulted in increased IFN-γ-positive cells in both HS and PD patients; however, the final proportion of IFN-γ-positive cells was higher in PD patients in comparison to HS (on average, + 39% in both PD-dn and PD-dt patients). Th2 polarizing conditions resulted in increased IL-4-positive cells in HS and PD-dn patients only, and as a consequence the proportion of IL-4-positive cells in PD-dt patients was less than in HS (on average, − 52%) (Fig. 4). Th17 polarizing conditions resulted in increased IL17-A-positive cells in HS only, thus the final proportion of IL17-A-positive cells in PD patients was less than in HS (on average, − 39% in PD-dn and − 41% in PD-dt patients) (Fig. 4).

### Study #2-CD4+ Treg in HS and PD patients.

#### Subjects

The study included 33 HS and 66 PD patients (Table 2), 30 PD-dn, and 36 PD-dt. In comparison to PD-dn patients, a larger fraction of PD-dt patients had H&Y stage 1.5 or more. Disease duration of PD-dt patients was 6.0±4.3 years (range 1–16 years). Complete blood counts of HS and PD patients were all within normal limits (Additional file 4: Table S3B). Compared to HS however PD-dn patients had less total lymphocytes (on average, about − 18%). PD-dt patients had total lymphocyte count lower than HS but higher than PD-dn patients, and the differences with both groups did not reach the statistical significance.

**Table 2:** Characteristics of HS and PD patients enrolled in study 2. Data are means ± SD unless otherwise indicated

	HS	PD-dn	PD-dt	P		
				HS vs PD-dn	HS vs PD-dt	PD-dn vs PD-dt
n	33	30	36			
Gender (M/F)	19/14	16/14	20/16	0.803	0.799	1.000
Age (years)	65.1 ± 11.4	68.2 ± 9.1	70.0 ± 8.1	0.241	0.133	0.698
UPDRS part III (score)		14.2 ± 7.0	17.3 ± 8.4			0.076
1–10 (*n*)		14	7			0.051
11–20 (*n*)		9	19			
> 20 (*n*)		7	10			
H&Y scale (stage)		1.4 ± 0.5	1.9 ± 0.8			0.002
1.0 (*n*)		17	10			0.014
1.5–2.0 (*n*)		12	17			
> 2.5 (*n*)		1	9			
LED (mg/day)			522.3 ± 356.5			
Drugs						
l-DOPA (*n*)			31^a^			
DA agonists (*n*)			17^b^			
Pramipexole (*n*)			8			
Ropinirole (*n*)			6			
Rotigotine (*n*)			3			
Rasagiline (*n*)			19			

^a^15 taking l-DOPA alone, and 16 taking l-DOPA + DA agents

^b^4 taking DA agonists alone (2) or with rasagiline (2), and 13 taking DA agonists + l-DOPA, without (10) or with rasagiline (3)

#### Circulating Treg

**Fig. 5:**
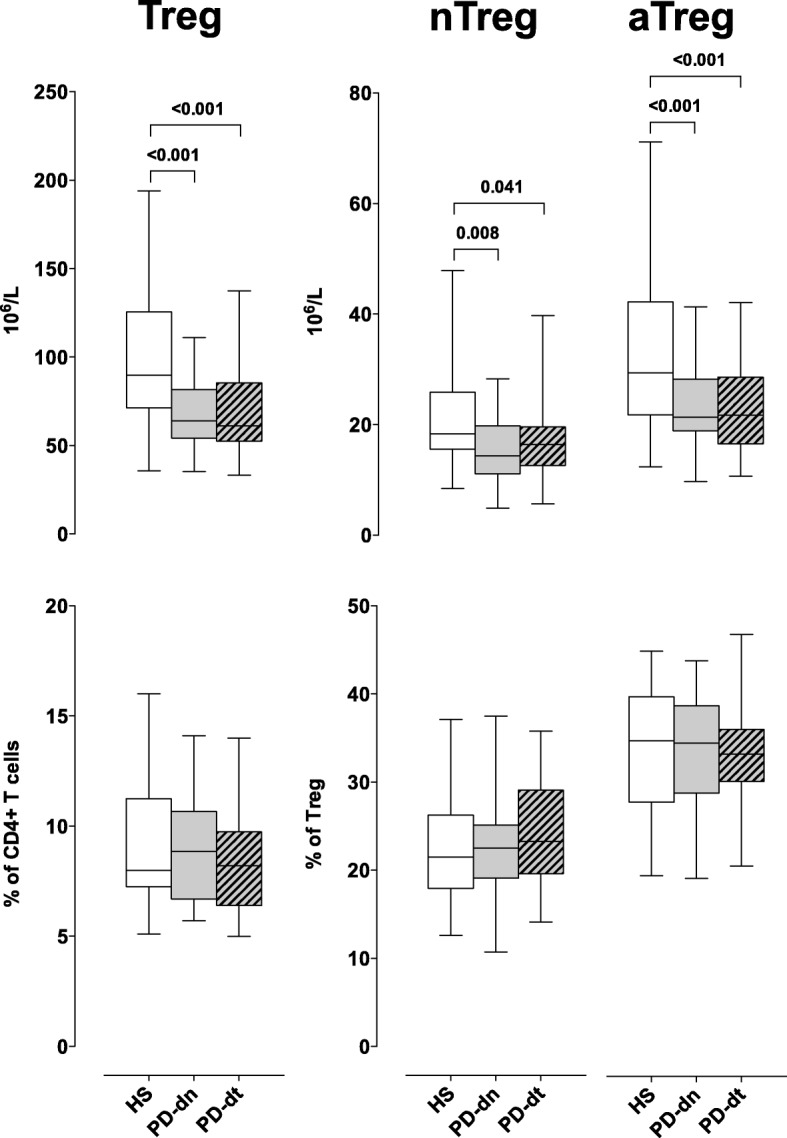
Treg cells in HS and PD patients. Cells are shown as absolute numbers (upper panels) and as percentage of total CD4+ T cells and of total Treg, respectively (lower panels). Data are medians with 25°–75° percentiles (boxes) and min–max values (whiskers)

In agreement with study #1, in both PD-dn and PD-dt patients CD4+ T cells were less than in HS, both as absolute counts and percentage of total lymphocytes, without any difference between PD-dn and PD-dt patients (Additional file 2: Figure S7). Both total circulating Treg as well as total nTreg and aTreg were less in PD-dn and PD-dt patients than in HS, while on the contrary their frequency was the same in HS and PD patients, due to reduced total CD4+ T cells. There was no difference between PD-dn and PD-dt patients (Fig. 5). In total Treg, the absolute count of D_2_-like DR+ cells was lower in both PD-dn and PD-dt patients in comparison to HS, while for D_1_-like DR+ cells, the difference with HS was significant for PD-dn patients only, even if there was no difference between PD-dn and PD-dt patients. Frequency of total DR+ Treg among CD4+ T cells was on the contrary the same in HS and PD patients (Additional file 2: Figure S8). A similar pattern of differences in DR+ cells between HS and PD patients was observed in nTreg, while for aTreg, the differences were only minor (Additional file 2: Figures S9 and S10).

#### Transcription factors mRNA levels in CD4+ T cells

The pattern of differences between HS and PD patients was the same as in study #1 (Additional file 2: Figure S11), with lower levels of *TBX21*, *STAT3*, *STAT4*, and *NR4A2*, and higher levels of *STAT6*, *GATA3*, and *FOXP3* in cells from PD patients. In study #2, *RORC* was lower not only in cells from PD-dt but also from PD-dn patients. *STAT1* expression was not different among HS, PD-dn, or PD-dt patients (Additional file 2: Figure S11).

#### Proliferation of Teff and inhibition by Treg

**Fig. 6:**
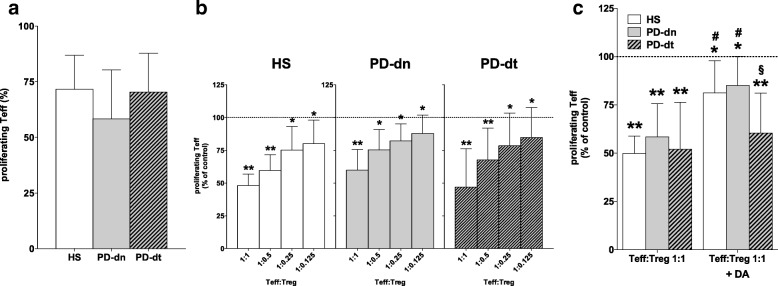
Treg-induced inhibition of Teff proliferation in HS and PD patients. Teff from HS and PD patients proliferated to a similar extent in the presence of PHA (**a**) and were concentration-dependently inhibited by Treg (**b**). Treg inhibition of Teff proliferation was reduced by DA 1 μM in cells from HS and PD-dn patients but not from PD-dt patients (**c**). Data are means ± SD of *n* = 9–17 subjects. *P < 0.05 and **P < 0.01 vs respective Teff alone; #*P* < 0.05 vs respective Teff:Treg 1:1; §*P* < 0.05 vs both HS and PD-dn

Teff proliferation in response to PHA was not different in HS, PD-dn, and PD-dt patients (Fig. 6a), and Treg effectively inhibited Teff proliferation to a similar extent in both HS and PD patients at all the Teff:Treg ratios tested (Fig. 6b). The inhibitory effect of Treg however was reduced by DA 1 μM only in HS and PD-dn but not in PD-dt patients (Fig. 6c).

#### Cytokine production by Teff and Treg

**Fig. 7:**
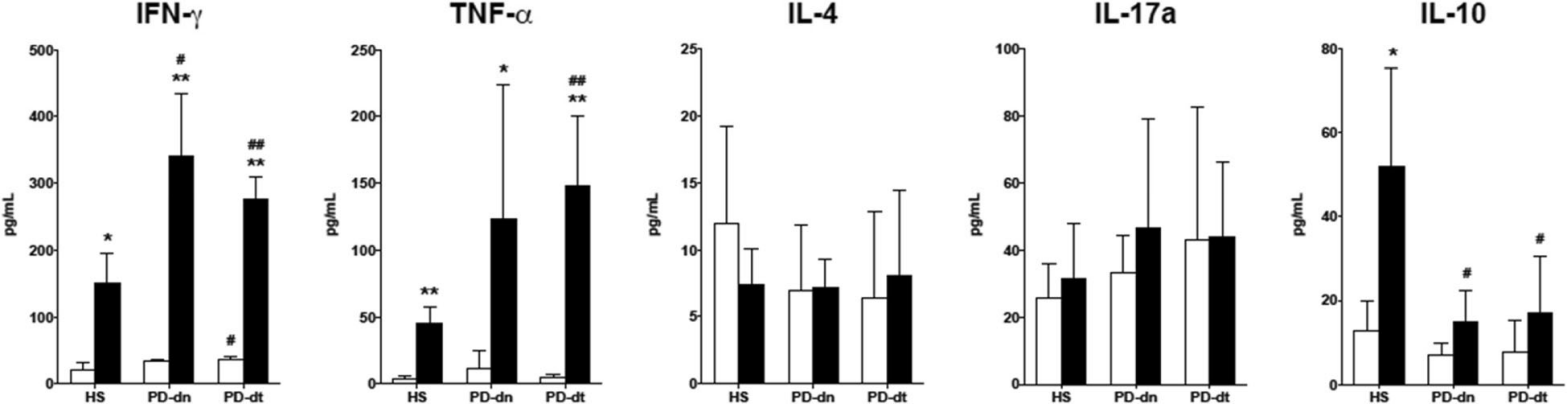
Cytokine production by Teff cells in HS and PD patients. Cytokines were measured in supernatants of resting cells (white columns) and of cells stimulated with PHA (black columns). Data are means ± SD of *n* = 4–9 subjects. *P < 0.05 and **P < 0.01 vs resting cells; # = *P* < 0.05 and ##*P* < 0.01 vs HS

Production of IFN-γ and TNF-α was stimulated by PHA in Teff from HS, PD-dn, and PD-dt patients. PHA-induced increase of IFN-γ and TNF-α production was however higher in cells from PD-dn and PD-dt patients compared to HS (on average, + 126% and + 179% in PD-dn, and + 82% and + 236% in PD-dt patients). There was no difference in IFN-γ or TNF-α production between cells from PD-dn and PD-dt patients (Fig. 7). Production of IL-4 and IL17A was not different in Teff from HS, PD-dn, and PD-dt patients, and was not affected by PHA (Fig. 7). Production of IL-10 was increased by PHA in cells from HS, but not from PD-dn and PD-dt patients (Fig. 7).

**Fig. 8:**
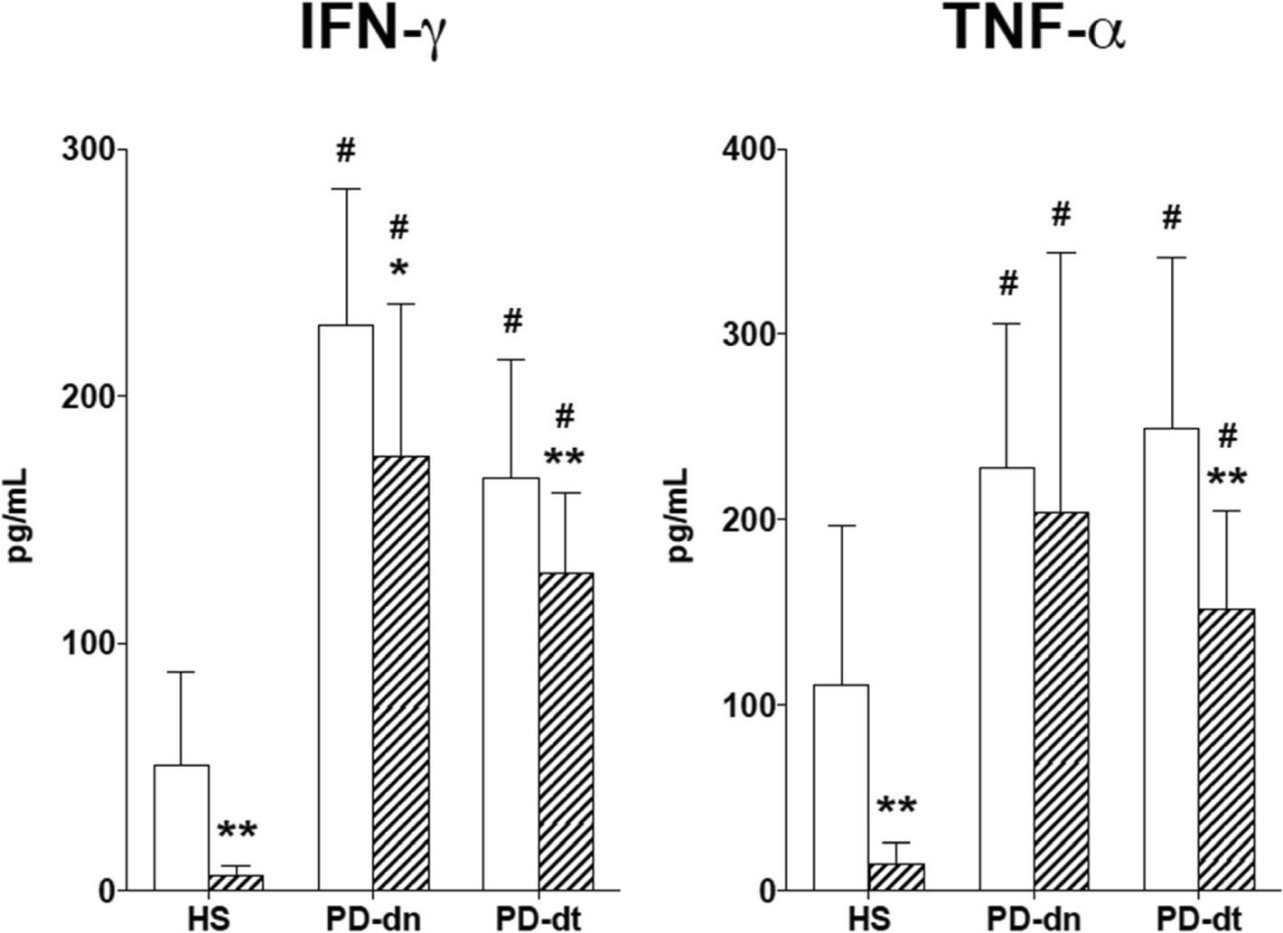
Teff production of IFN-γ and TNF-α and inhibition by Treg in HS and PD patients. Teff were stimulated with PHA alone (white columns) or in the presence of Treg 1:1 (hatched columns). Data are means ± SD of *n* = 6–10 subjects. *P < 0.05 and **P < 0.01 vs Teff alone; #*P* < 0.01 vs HS

Coincubation of Teff with Treg 1:1 reduced PHA-induced production of IFN-γ on average by 81% in cells from HS but only by 24 and 22% in cells from PD-dn and PD-dt patients. A similar pattern was observed with TNF-α (− 87% in cells from HS, − 16 and − 36% in cells from PD-dn and PD-dt patients) (Fig. 8). Production of IL-10 was not different in Treg from HS, PD-dn, and PD-dt patients (21.1±1.49, 14.6±7.2, and 19.4±6.2 pg/mL, respectively).

#### No correlations between circulating CD4+ T cells, DR expression, transcription factors mRNA levels, demographic, and clinical features of HS and PD patients

Circulating Th and Treg absolute counts or frequency, DR expression, and transcription factors mRNA levels in CD4+ T cells did not correlate with age in HS or with age, UPDRS part III score, and H&Y stage in PD-dn and PD-dt patients, or with disease duration and LED in PD-dt patients (data not shown).

## Discussion

Peripheral adaptive immunity in PD has been the subject of increasing interest over the last two decades, since in animal models of neurodegeneration T lymphocytes provide a major contribution to neuroinflammation and neuronal death, and targeting the peripheral immune system, e.g., by increasing Treg activity, may result in effective neuroprotection [32, 33]. Various studies indeed suggest the occurrence of peripheral immune changes in PD patients, including decreased CD4+/CD8+ T-cell ratios, fewer CD4 + CD25+ T cells, increased ratios of IFN-γ-producing to IL-4-producing T cells [34], and decreased CD4+ T lymphocytes and CD19+ B cells [35, 36]. A recent meta-analysis identified decreased numbers of CD3+ and CD4+ T lymphocytes as a consistent finding across 21 studies including 943 cases of PD [19]. These observations, together with the occurrence of T lymphocytes in postmortem brain specimens from PD patients [17, 18] as well as in a mouse model of PD [18], and with the markedly reduced dopaminergic neuronal death in mice lacking CD4+ T cells [18], strengthened the hypothesis that CD4+ T cells are crucial for neurodegeneration during PD, possibly through Th1-dependent mechanisms. We now provide detailed evidence about CD4+ T cell phenotypes count and frequency in the peripheral circulation of PD patients as well as about their functional profile. Of particular importance, we included in our studies both patients on dopaminergic substitution treatments as well as drug-naïve patients, who never received any dopaminergic drugs, thus allowing to assess any differences associated with current antiparkinson therapies. Main results can be summarized as follows: (i) PD patients, either drug-naïve or on dopaminergic drugs, have reduced circulating CD4+ T lymphocytes; (ii) CD4+ T cells reduction is accounted for mainly by reduced circulating Th2, Th17, Th1/17, and Treg, resulting in increased Th1/Th2 and Th1/Th17 ratio; (iii) lineage-specific transcription factors mRNA levels in CD4+ T cells from patients exhibit distinctive patterns; (iv) naïve CD4+ T cells isolated from blood of patients preferentially differentiate towards the Th1 lineage, and production of IFN-γ and of TNF-α by CD4+ T cells from patients is increased and maintained even in the presence of homologous Treg. Remarkably, only minor differences occurred between drug-naïve patients and patients on dopaminergic drugs, namely, in the latter group Treg insensitivity to DA.

### Decreased circulating CD4+ T cells

Decreased numbers of circulating CD4+ T lymphocytes in PD patients has been consistently described by several studies [19]. We previously reported that PD patients have on average 214.5 × 10^6^/L less lymphocytes in comparison to HS [23], a difference which is confirmed in the present investigation by both study #1 (− 244.0 × 10^6^/L) and study #2 (− 268.9 × 10^6^/L). Additional file 5: Table S4 summarizes the differences observed between PD patients and HS in our previous study [23] and in the present investigation. According to the whole picture, CD4+ T cells reduction in PD patients is accounted for by reduced Th2 (on average, − 23.7 × 10^6^/L), Th17 (− 31.1 × 10^6^/L), Th1/17 (− 33.8 × 10^6^/L), Treg (− 30.4 × 10^6^/L), and T naïve cells (− 117.2 × 10^6^/L), which altogether make a total of 236.2 × 10^6^/L less cells, an amount well matching with the global differences above reported for total CD4+ T cells. In this scenario, Th1 cell count is not different between PD patients and HS; however, in patients, the contextual reduction of the other phenotypes leads to their relative increase (on average, + 3.5% of total CD4+ T cells).

### The Th1 bias

**Fig. 9:**
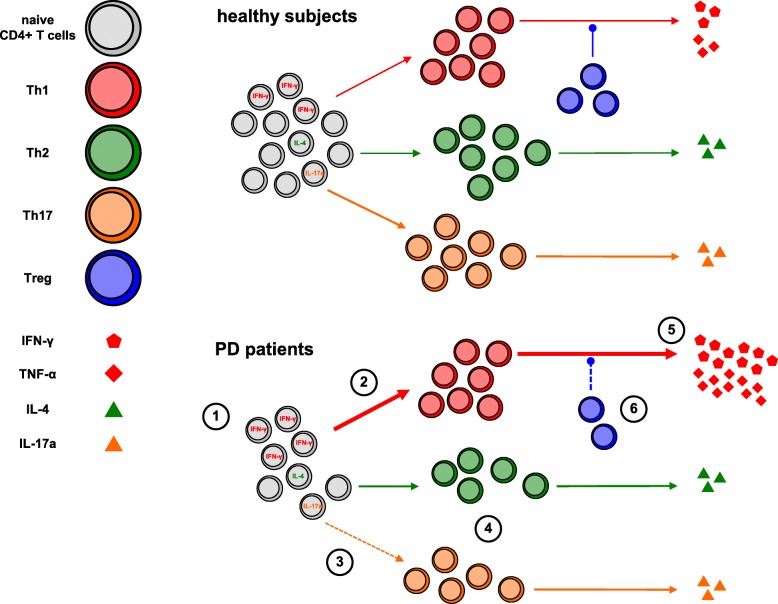
The Th1 bias in PD. Circulating CD4+ T naive cells in PD patients are reduced by about 30%; however, the proportion of IFN-γ-positive cells is increased (1). Differentiation towards the Th1 lineage is increased (2), while differentiation towards Th17 is impaired (3), and both Th2 and Th17 cells in blood are reduced on average by 20–30% (4). Production of IFN-γ and TNF-α by Th1 cells is strongly increased (5) and not impaired in the presence of Treg, which are also reduced by 30% in the circulation (6). Reduced production of IL-10 by CD4+ effector T cells, which likely contributes to amplify the Th1 bias, is not represented

Reduced circulating Th2, Th17, Th1/17, and Treg in PD patients leads to a relative increase of Th1 cells, which constitutes the basis for a possible Th1 bias, as also suggested by the increased Th1/Th2 and Th1/Th17 ratios in PD patients. Such bias is however made clear by results of in vitro functional tests, showing first of all a preferential differentiation of naïve CD4+ T cells from both drug-naïve patients and patients on dopaminergic drugs towards the Th1 lineage. Indeed, IFN-γ-positive Th0 cells were nearly two-fold in PD patients in comparison to HS, and Th1 polarizing conditions resulted in increased IFN-γ-positive cells in all PD patients, while Th2 polarizing conditions resulted in less IL-4-positive cells, at least in patients on dopaminergic drugs. Finally, Th17 polarizing conditions resulted in increased IL17-A-positive cells in HS, but not in PD patients either drug-naïve or on dopaminergic drugs. Preferential differentiation of naïve CD4+ T cells from PD patients towards the Th1 lineage suggests the involvement of homeostatic mechanisms driving naïve T cell differentiation [37]. Increased production of the Th1 cytokines IFN-γ and TNF-α by CD4+ effector T cells from PD patients also contributes to the Th1 bias. In comparison to cells from HS, IFN-γ, and TNF-α production in cells from either drug-naïve PD patients or from patients on dopaminergic drugs was indeed between two- and nearly three-fold higher. Finally, support to such Th1 bias in PD patients likely derives also from the lack of reduction of IFN-γ and TNF-α production by CD4+ effector T cells in the presence of Treg, as well as by the decreased production of IL-10 by CD4+ effector T cells themselves [38]. Figure 9 summarizes the differences occurring in circulating CD4+ T cells between HS and PD patients.

The occurrence of imbalances in the differentiation process towards the different CD4+ T cell lineages/phenotypes is indirectly supported also by the profound modifications of transcription factor genes expression in CD4+ T cells from PD patients in comparison to those from HS. In PD patients, CD4+ T cells expressed lower levels of *TBX21* and *STAT4*, which together with *STAT1* drive Th1 differentiation [39], as well as of *RORC* and *STAT3*, which regulate the differentiation towards the Th17 lineage [40], and higher levels of *STAT6* and *GATA3*, which are master regulators of Th2 development [41, 42]. *FOXP3*, which is centrally involved in the development and maintenance of the Treg phenotype [43], was upregulated, while *NR4A2*, encoding the orphan nuclear receptor Nurr1 which affects Treg cell development through activation of *FOXP3* [44], was downregulated. Transcription factor genes were assessed by means of mRNA levels, which however are not necessarily a homogeneous measure of gene transcript activity. Indeed, increased/decreased mRNA levels might mirror either increased/decreased transcription or decreased/increased translation (or any combination of either). Modification of transcription factors mRNA levels must be taken therefore as an indicator of their involvement in the disease, and not as a quantitative measure of transcription factors activation. For instance, modification of *TBX21* and *STAT4*, which mediate primarily IL-12-induced Th1 differentiation, but not of STAT1, which is involved in IFN-γ-dependent reinforcement of the Th1 phenotype [39], might therefore indicate a preferential involvement of IL-12-dependent pathways and a minor, if any, role of IFN-γ signaling in the increased Th1 differentiation and function of CD4+ T cells occurring in PD patients. Remarkably, such modifications occurred to the same extent in both drug-naïve patients as well as in patients on dopaminergic substitution treatments (Fig. 3 and Additional file 2: Figure S11), suggesting that modifications of the transcription factors network in CD4+ T cells occurs early in PD. The similar pattern and extent of modifications in all the patients and the absence of correlations between any of the transcription factors and patients characteristics, including age, age at onset, disease duration, LED, and UPDRS part III score, suggests that modification of CD4+ T cell differentiation mechanisms is independent from PD progression and severity, and insensitive to drug treatments.

### No relationship with dopaminergic substitution therapy or with PD patients’ characteristics

Our investigation provides phenotypic and functional evidence supporting a Th1 bias of circulating CD4+ T cells in PD patients, thus giving clinical relevance to preclinical observations in animal models of PD supporting the contribution of Th1-related mechanisms to neuroinflammation and neurodegeneration [45–47]. Our results also reveal a dysfunction of the Treg compartment, since Treg from PD patients inhibit the proliferation of effector CD4+ T cells to the same extent as those from HS, however they completely fail to reduce production of the Th1 cytokines IFN-γ and TNF-α. Dysfunctional Treg from PD patients were recently reported also by Saunders et al. (39), who however did not investigate the ability of these cells to modulate cytokine production. On the other side, reduced circulating Th17 and Th1/17 cells in PD patients, as well as no evidence for increased production of IL-17 by CD4+ T cells of patients, suggests a marginal if any role for this cell lineage in PD, despite evidence from animal models [46, 48]. Such peripheral Th1 bias, eventually amplified by dysfunctional Treg, occurs in both drug-naïve patients and in patients on dopaminergic drugs, suggesting that dopaminergic substitution treatments do not exert major effects on peripheral CD4+ T cells. Actually, in patients on dopaminergic drugs results show also insensitivity of Treg to dopaminergic functional inhibition. DA exerts a physiological inhibition of Treg suppressive activity [49], and resistance to DA in PD patients on dopaminergic drugs might possibly arise from desensitization of DA-operated pathways in these cells. The lack of major differences in DR expression on Treg between drug-naïve patients and patients on dopaminergic drugs however points to intracellular mechanisms. In any case, Treg resistance to DA might also imply that dopaminergic drugs would not affect Treg suppressive activity, a condition which at least in principle could be beneficial in PD.

### Possible origin and implications of the Th1 bias

The major question posed by this finding is whether such immune profile represents a preexisting milieu favoring subsequent neuroinflammation, or whether it arises as a consequence of peripheral leakage of CNS-derived neoantigens, such as modified α-synuclein within Lewy bodies released from dying or dead dopaminergic neurons, as suggested by animal models of neurodegeneration [50]. In this regard, careful consideration might be deserved by emerging evidence about the role of the immune system as key regulator of the influence on brain development and homeostasis exerted by the gut microbiome [51]. A recent study compared the fecal microbiomes of PD patients and controls, showing in feces of PD patients nearly 80% decreased abundance of *Prevotellaceae* and increased abundance of other families including *Lactobacillaceae* [52]. Although how the intestinal microbiome contributes to development, homeostasis, and activation of the immune system is still largely unknown, it is remarkable that *Prevotellaceae* abundance may be associated with increased Th17-mediated mucosal inflammation, in line with their ability to drive Th17 immune responses in vitro [53]. On the other side, several lines of evidence indicate that *Lactobacillaceae* may induce Th1-type immune responses [54]. Taking together available evidence, it might be speculated that modifications of the intestinal microbiome in PD is a major driver for the development of immune traits such as reduced Th17 cells and Th1-biased immunity. Indeed, clinical and pathological evidence support the hypothesis that PD starts in the gut, where α-synuclein-related neurodegeneration of the enteric nervous system is a frequent and early manifestation of PD (reviewed in [55]). Presentation of α-synuclein to lymphocytes homing into the gut-associated lymphoid tissue, in the context of a disbiotic intestinal milieu, might well promote α-synuclein antigenic potential, driving detrimental T cell responses in PD patients [56]. The peripheral immune signature of PD might thus arise in the gut from a complex interaction among intestinal microbioma, the enteric nervous system and the immune system, with the possible influence of environmental factors eventually affecting any of the three actors.

Defining the time sequence of peripheral immune activation with respect to central neuroinflammation would be indeed of key importance to establish the potential of peripheral immunity as target for novel therapeutic interventions aimed at preventing neurodegeneration. A major obstacle in this regard is however the lack of solid biomarkers for the early identification of very initial preclinical conditions which will over the years progress to clinically established PD. Interestingly, constipation is a very common occurrence in PD and evidence suggests that it may even represent a major risk factor for subsequent PD development even several years later (see e.g., [57]). Indeed, circumstantial evidence suggests that chronic constipation might be associated with immune activation [58]. Would it be possible that among subjects with severe constipation, those subsequently developing PD would be preferentially those with the most prominent proinflammatory immune profile? A prospective study including an adequate sample of subjects with severe chronic constipation periodically assessed for their peripheral immune profile in comparison to their neurological conditions might possibly contribute to answer these questions.

### Limits of the investigation

The main limitations of our studies are the cross-sectional design and the inability to perform all the assays in all the subjects. A longitudinal design would allow to study the same subjects over a period of time, detecting changes at both group and individual level, thus allowing to establish a sequence of events. Our project, aimed at investigating peripheral adaptive immunity on PD, started however with a set of cross-sectional studies as this kind of studies can be done more quickly than longitudinal studies, and allowed us to establish main differences and similarities between PD patients and HS, as well as between drug-naïve patients and patients on dopaminergic drugs. Based on results, we already started a longitudinal protocol including all the drug-naïve patients so far recruited, as well as additional ones that we are presently recruiting, to assess any changes in the phenotypic and functional CD4+ T cells profile over several years. Planned evaluations include at present both 2- and 4-year follow-up.

Inability to perform all the assays in all the subjects and the subsequent need to include in the present investigation two study protocols, the first focusing on Th1/Th2/Th17 and the second one on Treg, were due mainly to the time sequence of validation of the different flow cytometric panels and of the in vitro models for the functional assessment of CD4+ T cell subsets. Requirement of significant amounts of blood for in vitro models was another factor which limited the possibility to assess all the cellular phenotypes and functions in all the subjects. Nonetheless, performing two separate studies also allowed us to check the reproducibility of many results. Indeed, total leukocyte and specific CD4+ T cell counts, as well as transcription factors expression in CD4+ T cells, were performed in all the subjects and provided consistent results across the studies, as shown in Additional file 4: Table S3A and B for total leukocytes, in Fig. 1 and in Additional file 2: Figure S7 for CD4+ T cell counts, and in Fig. 3 and in Additional file 2: Figure S11 for transcription factors. An additional limitation is the assessment of dopaminergic substitution treatment only at the time of enrolment. Regarding dopaminergic drugs, although we used LED [27] to obtain a homogenous measurement of treatment intensity, we were unable to assess treatment duration and changes in time. Such limitation will be overcome only using a longitudinal study design.

## Conclusions

The results of our investigation provide for the first time a comprehensive and detailed account of a complex phenotypic and functional Th1-biased adaptive immune response occurring in peripheral blood of PD patients. Reduction of CD4+ T cells as also reported in previous studies might apparently suggest impairment of peripheral immunity. The present results, however, including detailed analysis of CD4+ T cell subsets coupled to their functional characterization, allow now detailed understanding of many subtle and concurrent changes affecting several components of the CD4+ T cell compartment, leading as a whole to a prominence of Th1 immunity. In particular, reported functional responses specific to the different CD4+ T cell subsets represent suitable biomarkers for both the ex vivo/in vitro assessment of candidate peripheral immune system-targeted treatments for PD, as well as for the assessment of their effects in clinical trials. Recently, β_2_-adrenoceptors (β_2_-AR) were suggested as brand novel therapeutic targets in PD, based on their ability to modulate α-synuclein gene expression in human cells and to abrogate neurodegeneration in the MPTP mouse model of PD, and considering the association in the Norwegian population between use of the β_2_-AR agonist salbutamol and of the β_2_-AR antagonist propranolol with, respectively, reduced and increased risk of PD [59]. Remarkably, β_2_-AR are the main interface between sympathoadrenergic nerves and immune cells [60–62], and stimulation of β_2_-AR on CD4+ T lymphocytes inhibits Th1 and stimulates Th2 responses [60, 61] and possibly enhances Treg suppressive functions [63]. β_2_-AR agonists represent therefore obvious candidates to be tested on CD4+ T cells from PD patients for their ability to correct dysfunctional responses such as Th1-biased differentiation of CD4+ naïve T cells and increased production of Th1 cytokines insensitive to Treg inhibition. Any drugs which would result effective in such in vitro testings might well be candidated for therapeutic trials in PD patients as add-ons to conventional dopaminergic substitution treatments.

## Additional files

Additional file 1: **Table S1.** List of ab used in flow cytometric assays. (DOCX 19 kb)

Additional file 2: **Figure S1.** CD4+ Th cells and DR expression in whole blood. Gating strategy used to identify DR + CD4+ T lymphocytes. **Figure S2.** CD4+ Treg cells and DR expression in whole blood. Gating strategy used to identify DR + CD4+ Treg cells. ** Figure S3.** DR expression on Th1 cells in HS and PD patients. **Figure S4.** DR expression on Th2 cells in HS and PD patients. **Figure S5.** DR expression on Th17 cells in HS and PD patients. **Figure S6.** DR expression on Th1/17 cells in HS and PD patients. **Figure S7.** CD4+ T cells in HS and PD patients enrolled in study #2. **Figure S8.** DR expression on Treg cells in HS and PD patients. **Figure S9.** DR expression on nTreg cells in HS and PD patients. **Figure S10.** DR expression on aTreg cells in HS and PD patients. **Figure S11.** Transcription factors mRNA levels in CD4+ T cells of HS and PD patients enrolled in study #2. (PPTX 10597 kb)

Additional file 3: **Table S2.** Real-time PCR conditions. (DOCX 24 kb)

Additional file 4: **Table S3.** Complete blood count in HS and PD patients. Data are means ± SD unless otherwise indicated. (DOCX 45 kb)

Additional file 5: **Table S4.** Lymphocyte count, comparison between HS and PD patients. Data are means ± SD unless otherwise indicated. Differences are indicated only when statistically significant, and are reported as the mean differences (with 95% confidence interval) between the means. (DOCX 48 kb)

## Abbreviations

BSA: Bovine serum albumin
DA: Dopamine
DR: Dopaminergic receptor
H&Y: Hoehn and Yahr scale
HS: Healthy subjects
LED: l-DOPA equivalent dose
PBMC: Peripheral blood mononuclear cells
PD: Parkinson’s disease
PD-dn: Patients with idiopathic PD either drug-naïve
PD-dt: PD patients on antiparkinson drug treatment
Teff: T effector cells
Th: T helper cells
Treg: T regulatory cells
