# Supplementary material for: Parkinson’s disease patients have a complex phenotypic and functional Th1 bias: cross-sectional studies of CD4+ Th1/Th2/T17 and Treg in drug-naïve and drug-treated patients
Source: J Neuroinflammation. 2018 Jul 12;15:205. doi: 10.1186/s12974-018-1248-8 (PMC6044047; doi:10.1186/s12974-018-1248-8)
Supplement: Supplementary file 1 — Table S1. List of ab used in flow cytometric assays. (DOCX 19 kb) [file 12974_2018_1248_MOESM1_ESM.docx]

**Table S1.** List of ab used in flow cytometric assays.

| **Target** | **Supplier** | **Species** | **Clone; Isotype** | **Conjugation** | **Volume**  **(µL/tube)** | **Reference** |
| --- | --- | --- | --- | --- | --- | --- |
| **Panel for T helper cells** | | | | | | |
| Human CD4 | Becton Dickinson-Italy | mouse | RPA-T4; IgG1, k | APCCy7 | 2.5 | 557871 |
| Human CD183 (CXCR3) | Becton Dickinson-Italy | mouse | 1C6/CXCR3; IgG1, k | AF488 | 5 | 558047 |
| Human CD194 (CCR4) | Becton Dickinson-Italy | mouse | 1G1; IgG1, k | PECy7 | 2.5 | 557864 |
| Human CD196 (CCR6) | Biolegend-Campoverde | mouse | G034E3; IgG2b, k | PerCPCy5.5 | 5 | 353406 |
| **Panel for T regulatory cells** | | | | | | |
| Human CD4 | Becton Dickinson-Italy | mouse | RPA-T4; IgG1, k | APCCy7 | 2.5 | 557871 |
| Human CD25 | Becton Dickinson-Italy | mouse | M-A251; IgG1,k | PE-Cy7 | 2.5 | 557741 |
| Human CD127 | Becton Dickinson-Italy | mouse | HIL-7R-M21; IgG1, k | PerCPCy5.5 | 2.5 | 560551 |
| Human CD45RA | Biolegend-Campoverde | mouse | HI100; IgG2b, k | FITC | 2.5 | 304106 |
| **Antibodies for DR** | | | | | | |
| Human DR D_1_ | Merck-Millipore | Rabbit | IgG, polyclonal | n/a | 10^a^ | 324390 |
| Human DR D_5_ | Merck-Millipore | Rabbit | IgG, polyclonal | n/a | 10^a^ | 324408 |
| Human DR D_2_ | LifeSpan-Space | Rabbit | IgG, polyclonal | n/a | 10^a^ | LS-C22924 |
| Human DR D_3_ | Merck-Millipore | Rabbit | IgG, polyclonal | n/a | 10^a^ | 324402 |
| Human DR D_4_ | LifeSpan-Space | Rabbit | IgG, polyclonal | n/a | 10^a^ | LS-C22938 |
| PEGAR ab | R&D System, Space Import Exp | Goat | IgG, polyclonal | PE | undiluted | F0110 |

a = from a stock solution prediluted 1:10.
