# Supplementary material for: Parkinson’s disease patients have a complex phenotypic and functional Th1 bias: cross-sectional studies of CD4+ Th1/Th2/T17 and Treg in drug-naïve and drug-treated patients
Source: J Neuroinflammation. 2018 Jul 12;15:205. doi: 10.1186/s12974-018-1248-8 (PMC6044047; doi:10.1186/s12974-018-1248-8)
Supplement: Supplementary file 2 — Figure S1. CD4+ Th cells and DR expression in whole blood. Gating strategy used to identify DR + CD4+ T lymphocytes. Figure S2. CD4+ Treg cells and DR expression in whole blood. Gating strategy used to identify DR + CD4+ Treg cells. Figure S3. DR expression on Th1 cells in HS and PD patients. Figure S4. DR expression on Th2 cells in HS and PD patients. Figure S5. DR expression on Th17 cells in HS and PD patients. Figure S6. DR expression on Th1/17 cells in HS and PD patients. Figure S7. CD4+ T cells in HS and PD patients enrolled in study #2. Figure S8. DR expression on Treg cells in HS and PD patients. Figure S9. DR expression on nTreg cells in HS and PD patients. Figure S10. DR expression on aTreg cells in HS and PD patients. Figure S11. Transcription factors mRNA levels in CD4+ T cells of HS and PD patients enrolled in study #2. (PPTX 10597 kb) [file 12974_2018_1248_MOESM2_ESM.pptx]

## Slide 1
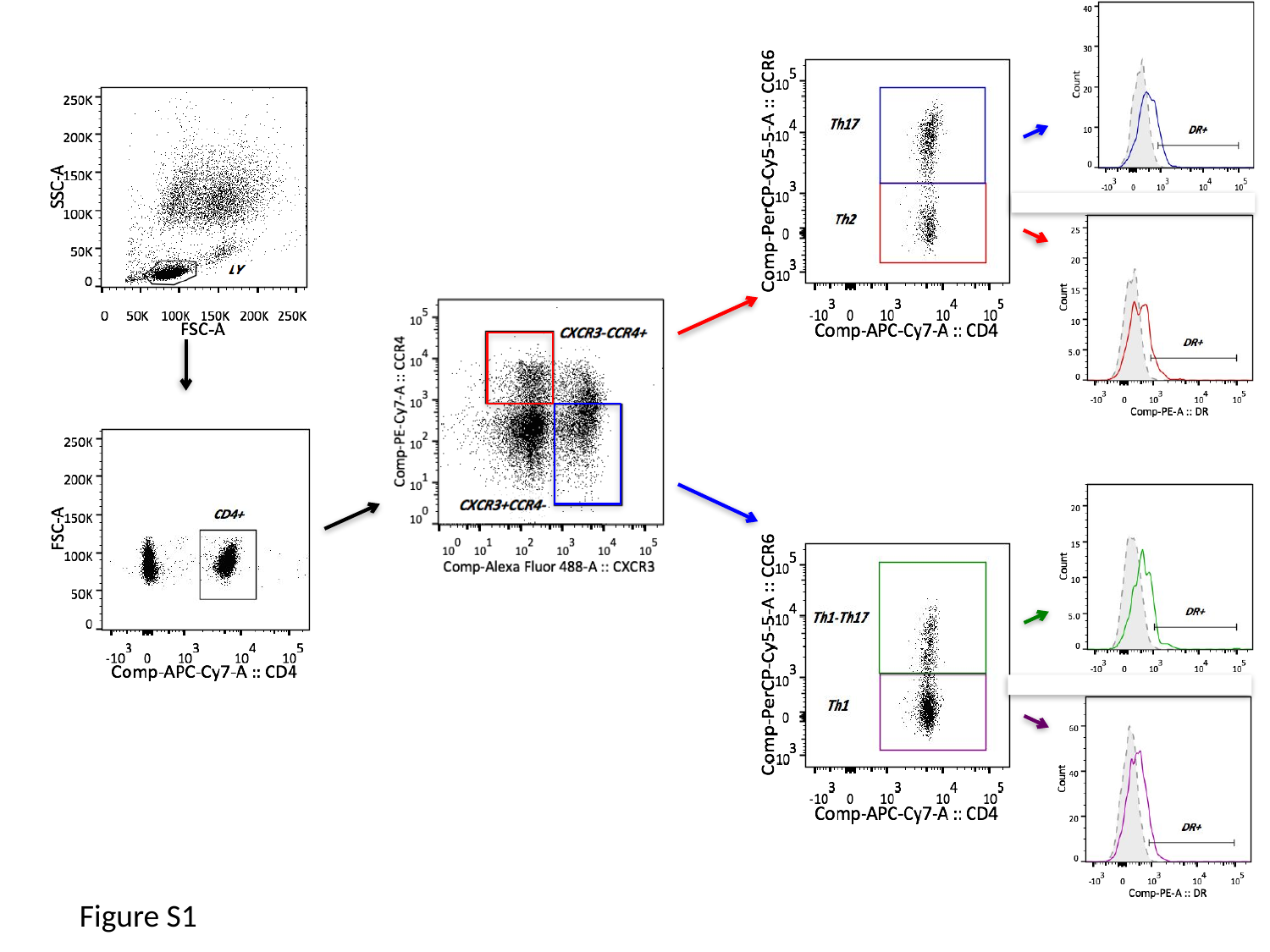

Figure S1

## Slide 2
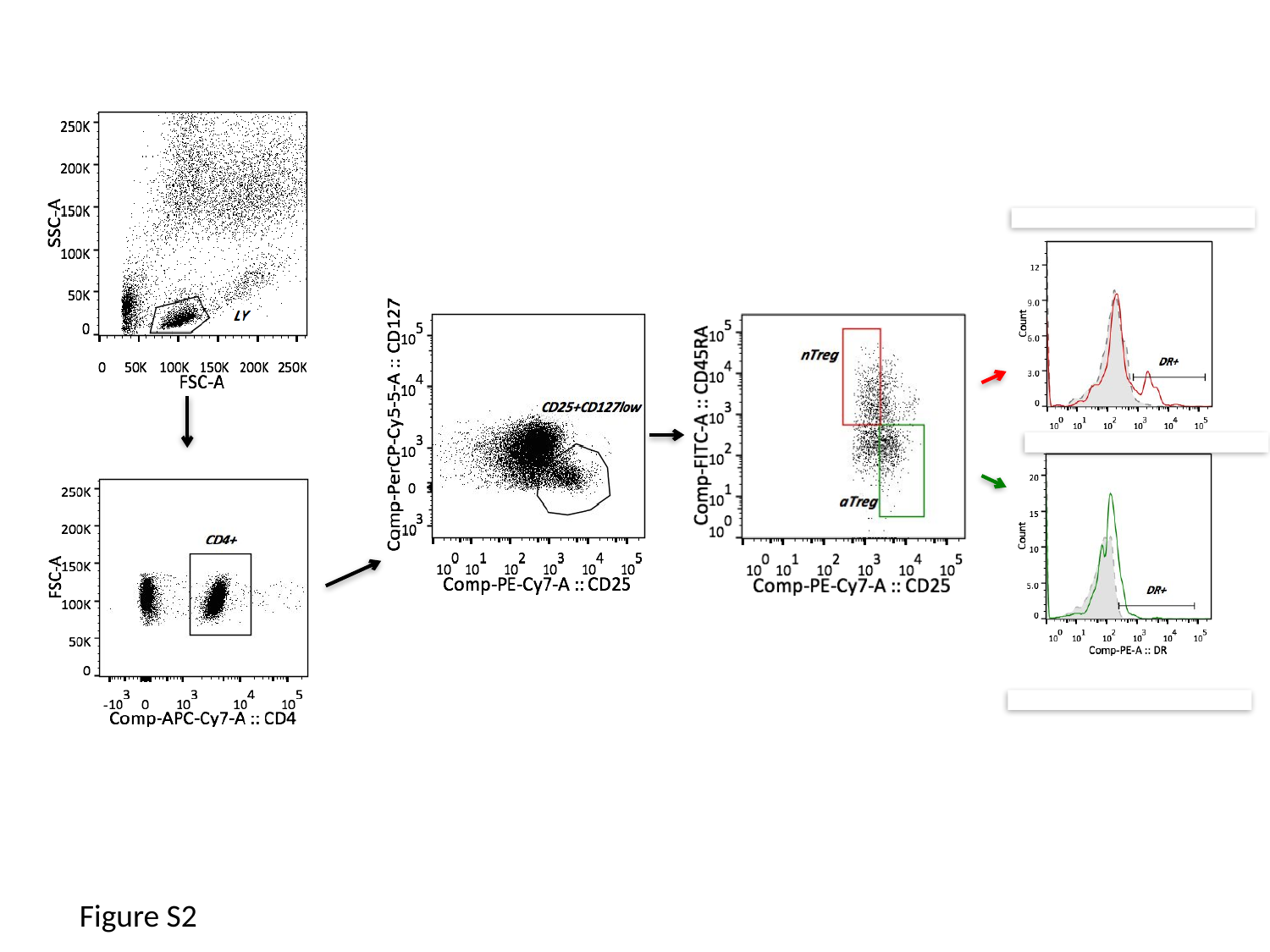

Figure S2

## Slide 3
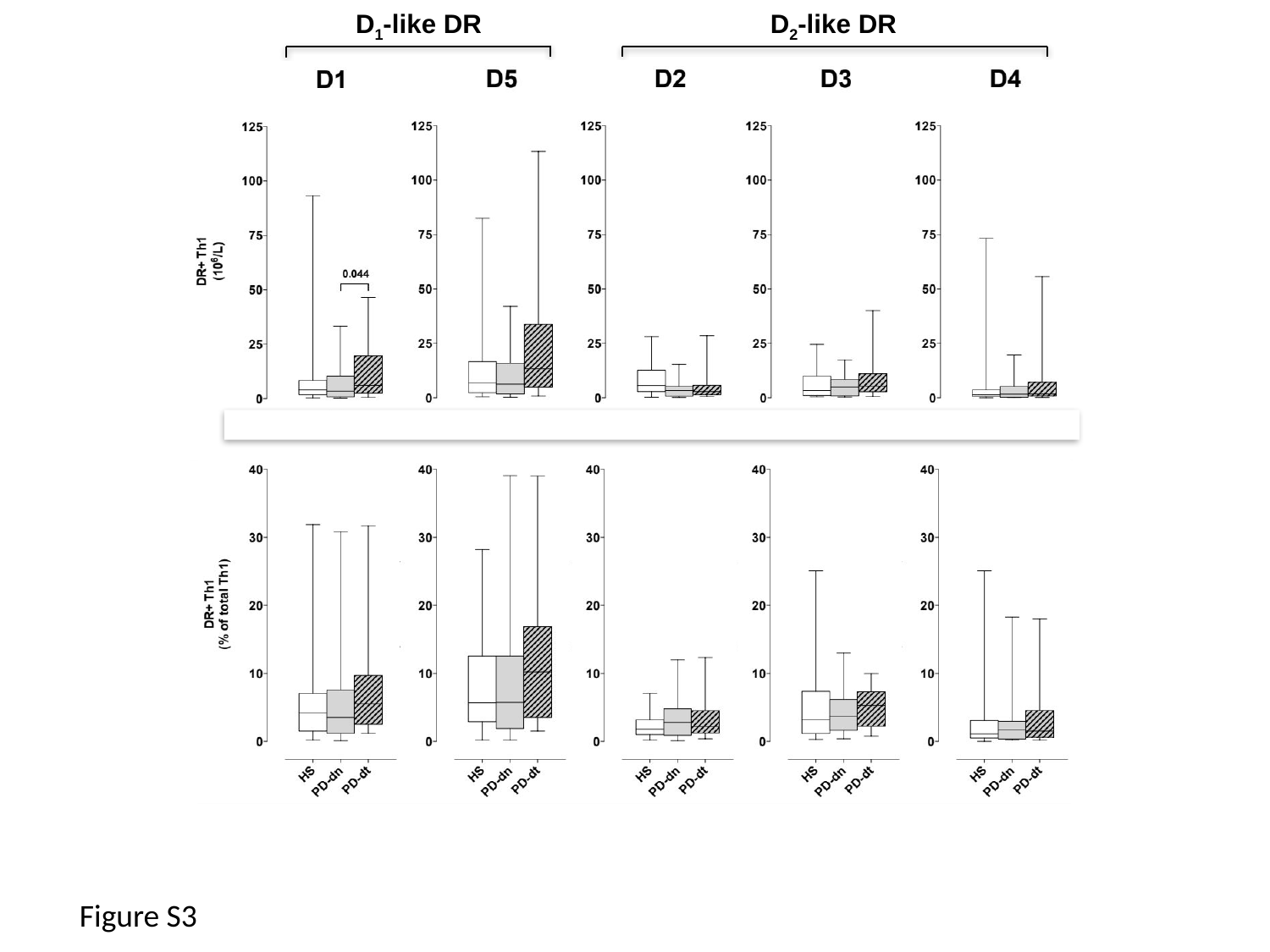

D2-like DR
D1-like DR
Figure S3

## Slide 4
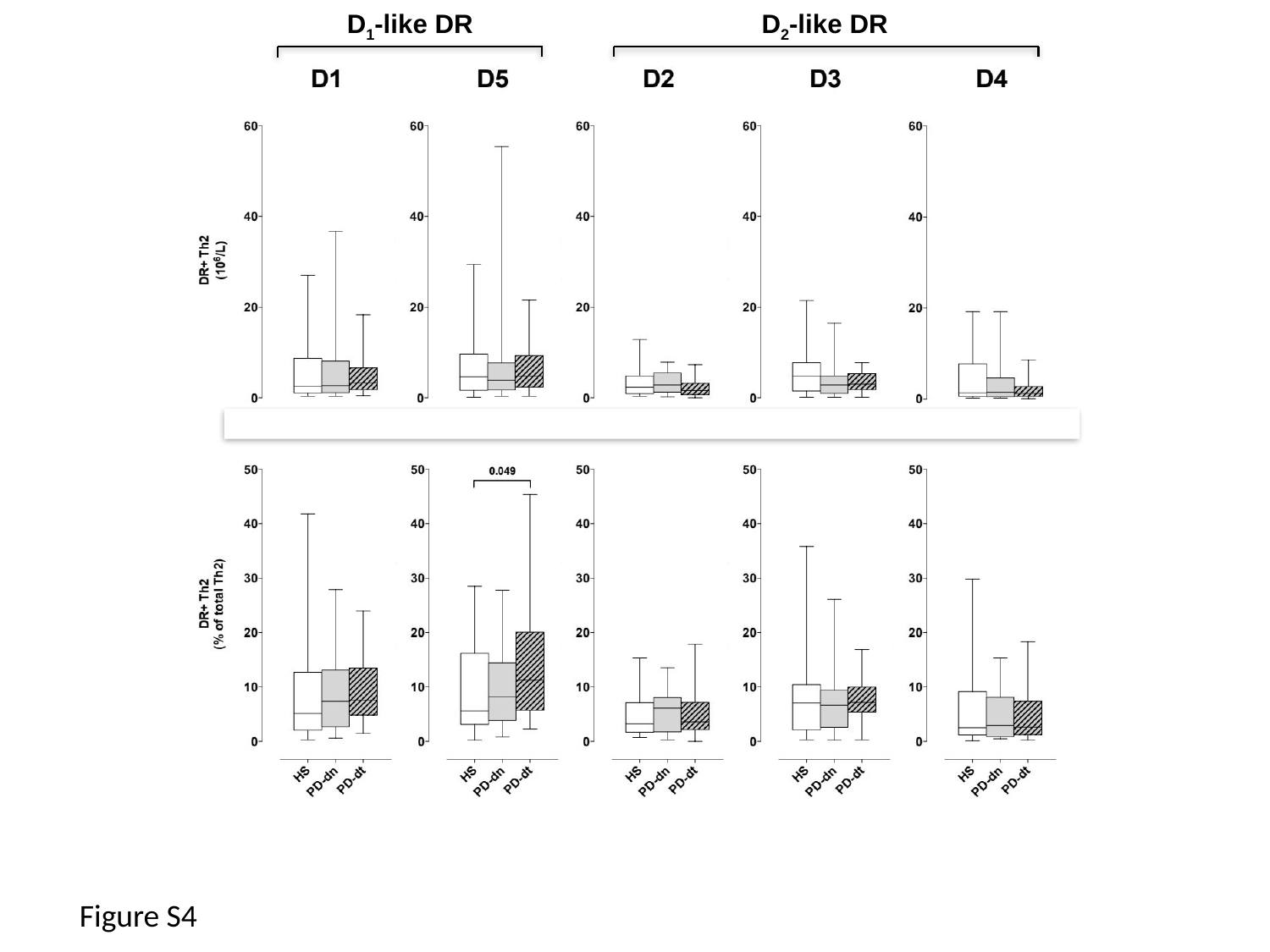

D2-like DR
D1-like DR
Figure S4

## Slide 5
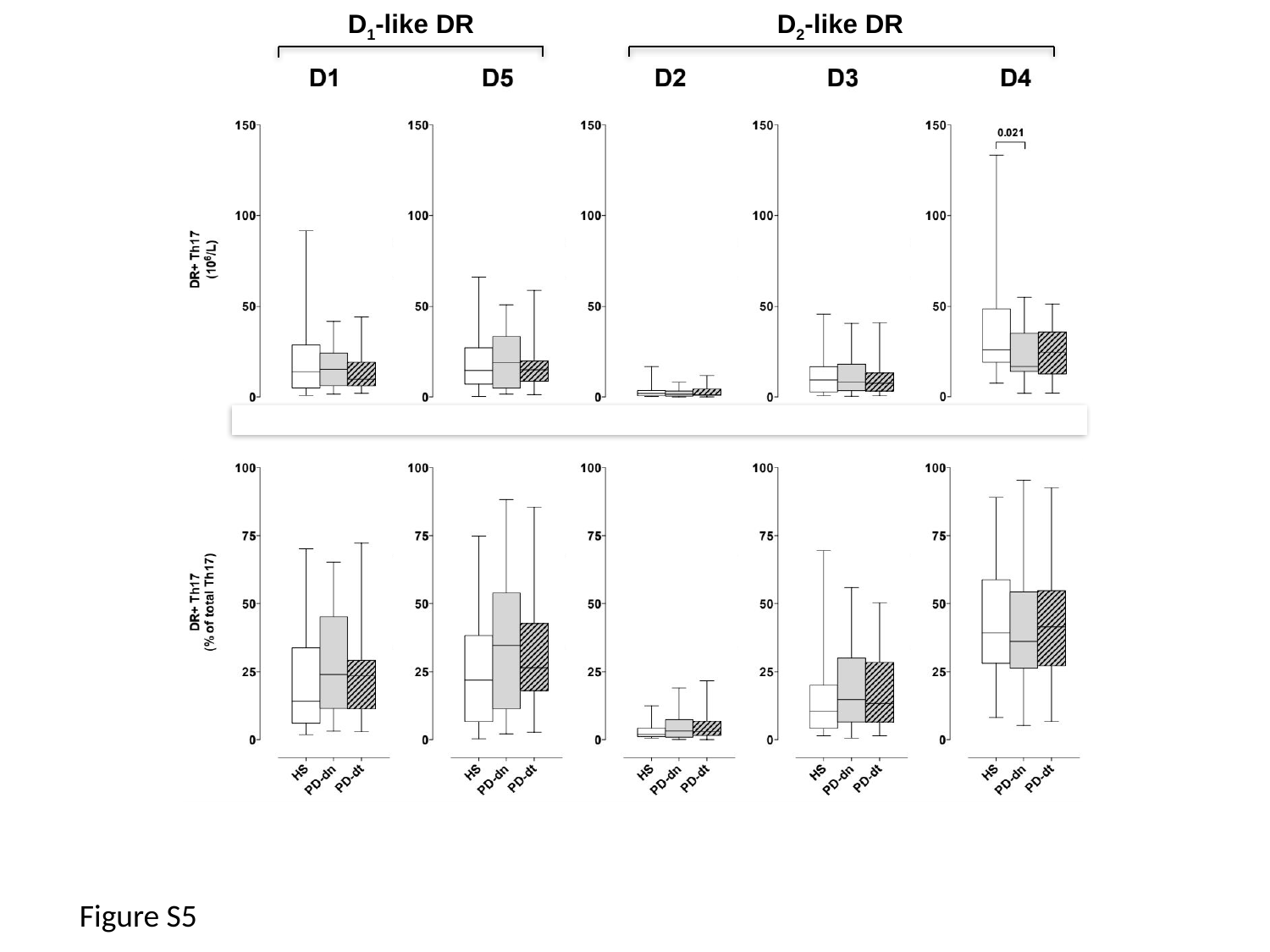

D2-like DR
D1-like DR
Figure S5

## Slide 6
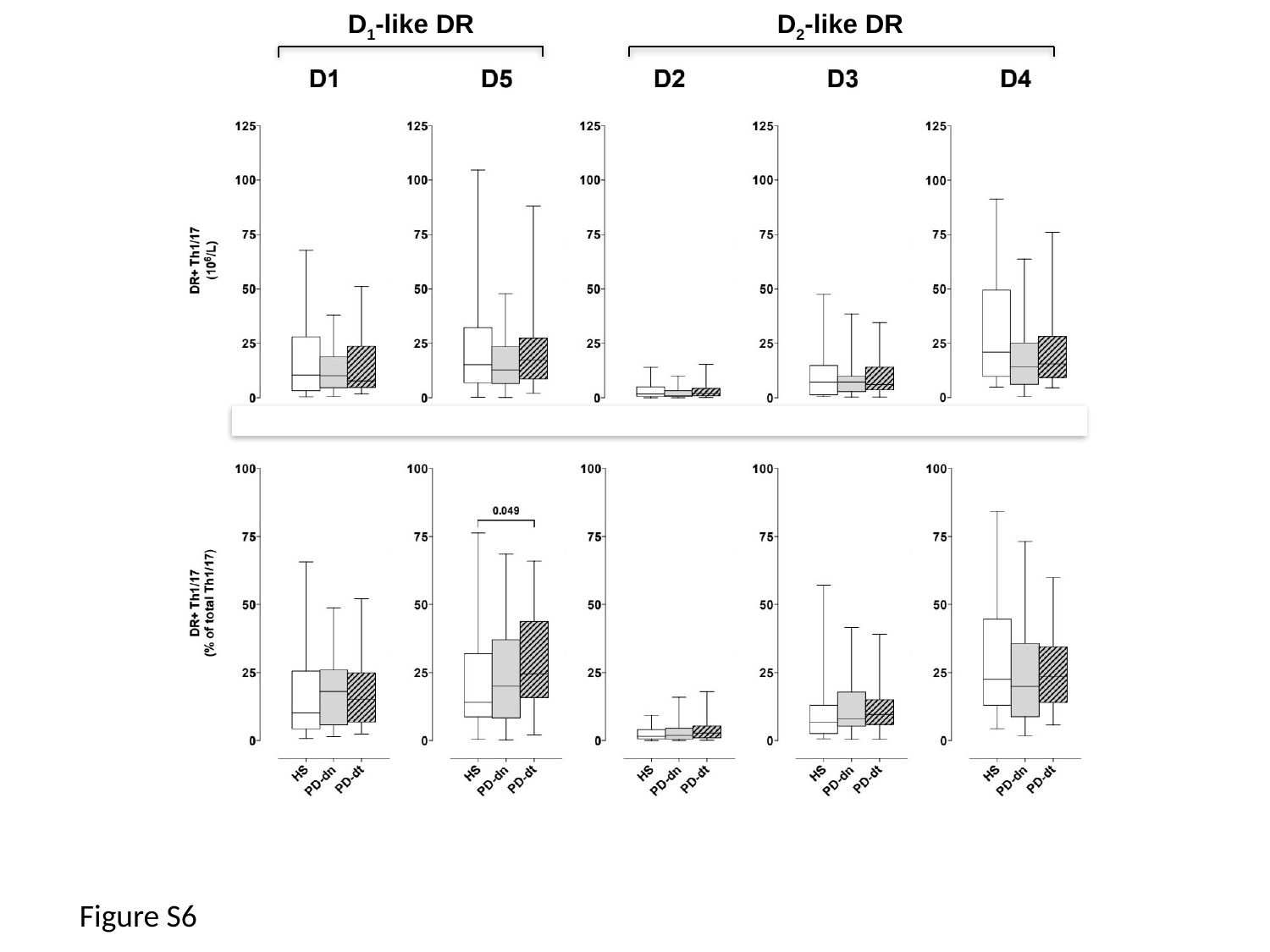

D2-like DR
D1-like DR
Figure S6

## Slide 7
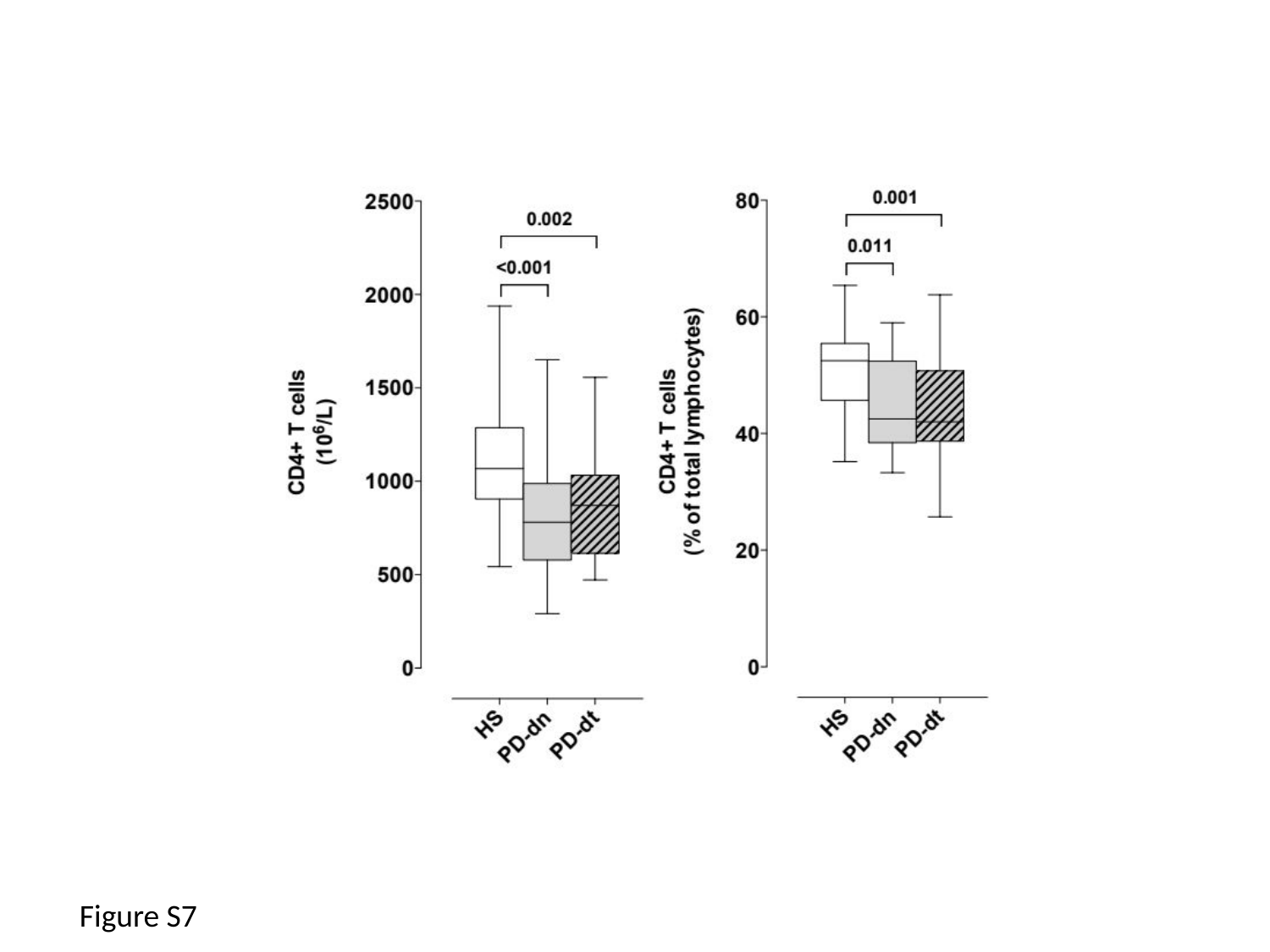

Figure S7

## Slide 8
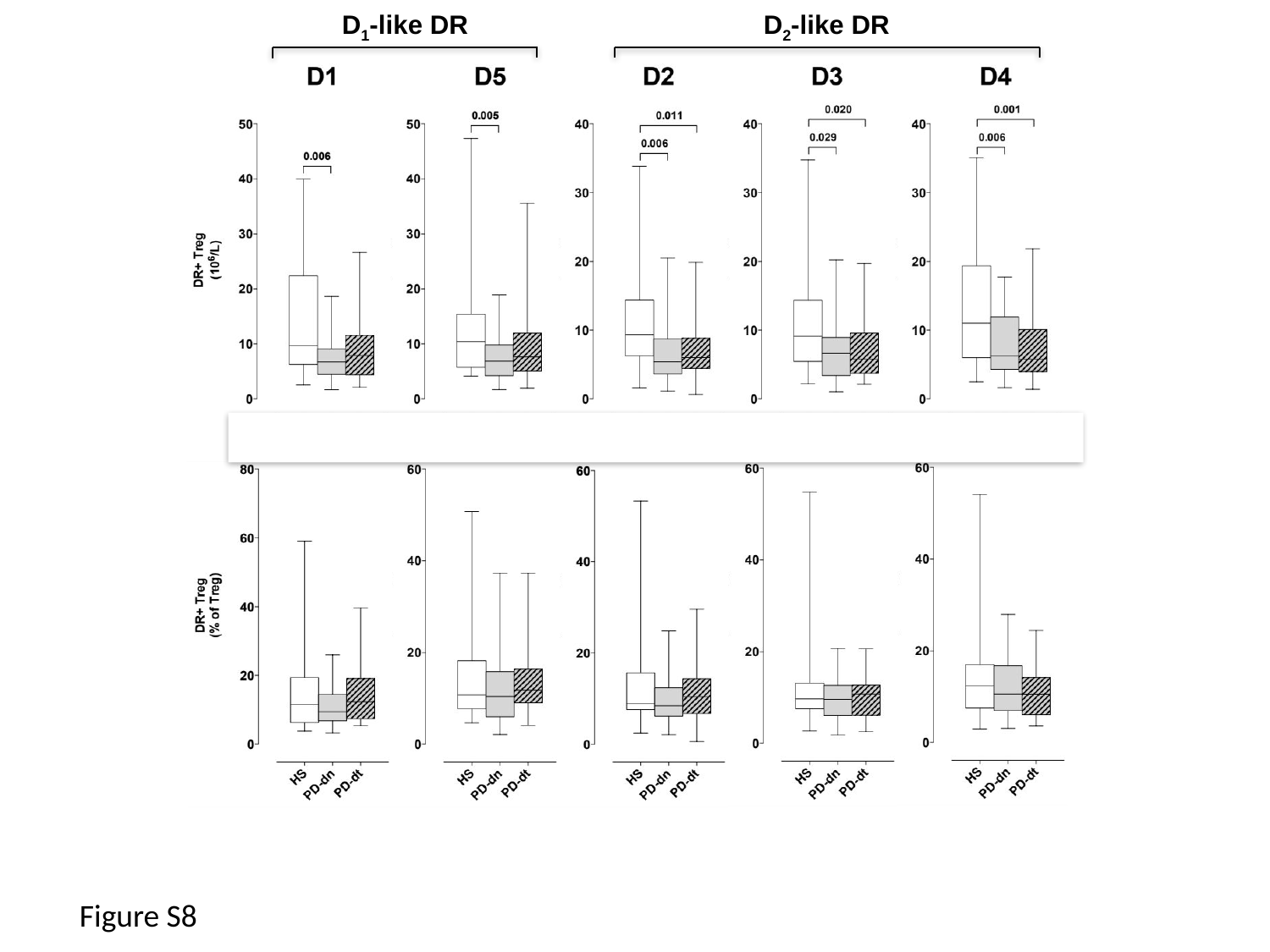

D2-like DR
D1-like DR
Figure S8

## Slide 9
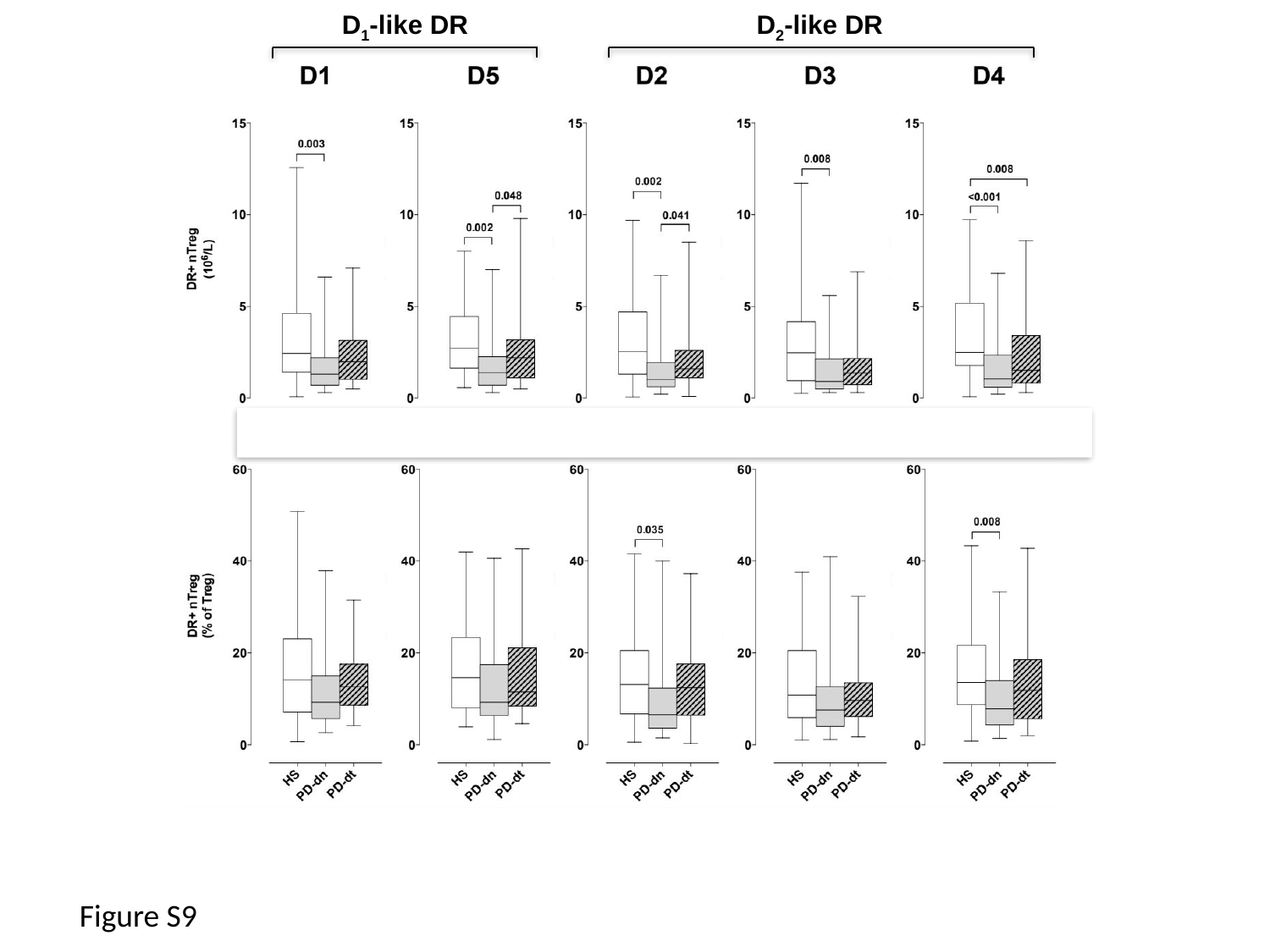

D2-like DR
D1-like DR
Figure S9

## Slide 10
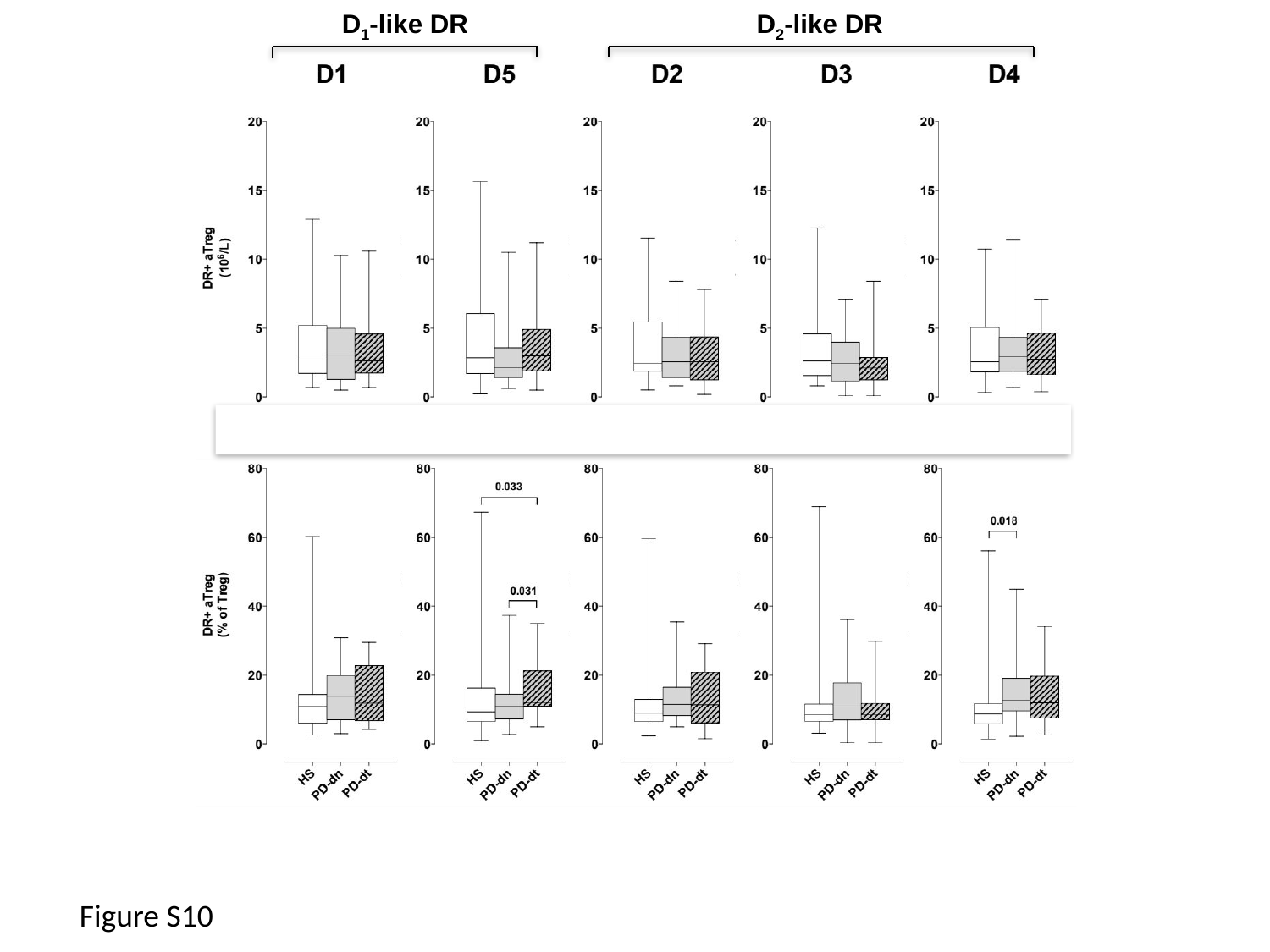

D2-like DR
D1-like DR
Figure S10

## Slide 11
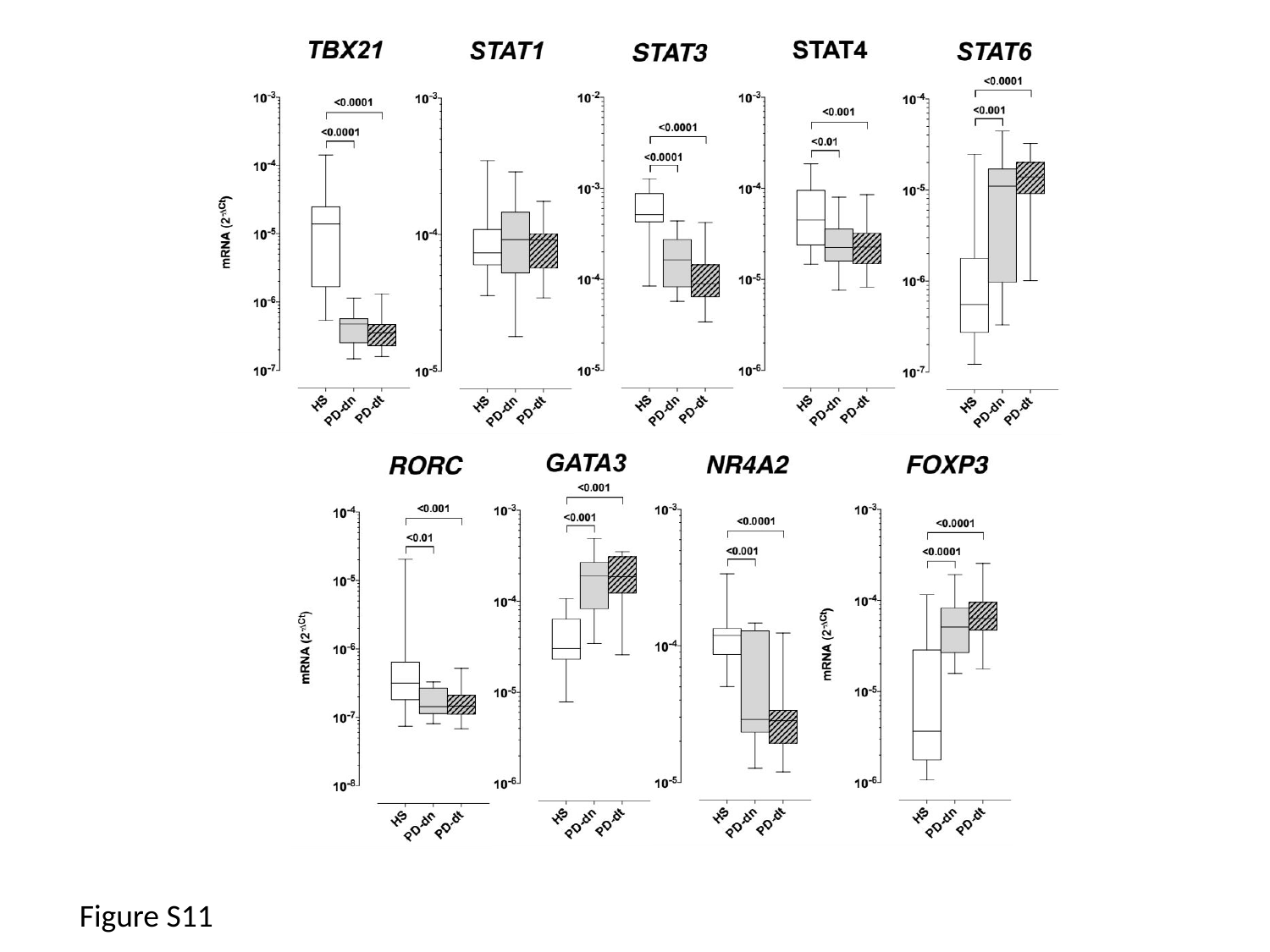

Figure S11
