# Supplementary material for: Parkinson’s disease patients have a complex phenotypic and functional Th1 bias: cross-sectional studies of CD4+ Th1/Th2/T17 and Treg in drug-naïve and drug-treated patients
Source: J Neuroinflammation. 2018 Jul 12;15:205. doi: 10.1186/s12974-018-1248-8 (PMC6044047; doi:10.1186/s12974-018-1248-8)
Supplement: Supplementary file 3 — Table S2. Real-time PCR conditions. (DOCX 24 kb) [file 12974_2018_1248_MOESM3_ESM.docx]

**Table S2.** Real-Time PCR conditions.

| **Gene** | **UniGene**  **ID** | **Interrogated sequences**  *RefSeq/GenBank mRNA* | **Detected coding transcripts** | **Amplicon context sequence** | **Chromosome**  **location** | **Amplicon**  **length** | **Annealing temperature**  (°C) | **Efficiency**  (%) |
| --- | --- | --- | --- | --- | --- | --- | --- | --- |
| *TBX21* | Hs.272409 | NC_000017.10  NG_012166.1  NT_010783.15 | ENST00000177694 | GTTTTATAACTATTTTCCCAACTGAGCAGATGACATGATGAAAGGAACAGAAACAGTGTTATTAGGTTGGAGGACACCGACTAATTTGGGAAACGGATGAAG | 17:45822708-45822809 | 72 | 60 | 97 |
| *STAT1* | Hs.731486 | NC_000002.11  NG_008294.1  NT_005403.17 | ENST00000361099  ENST00000409465  ENST00000392322  ENST00000392323  ENST00000424722  ENST00000454414  ENST00000432058  ENST00000540176 | CCAGTCTTGCTTTTCTAACCACTGTGCCAGGTACTGTCTGATTTCCATGGGAAAACTGTCATCATAAAGCTGGTGAACCTGCTCCAGGAATTTTGAGTCAAGCTGCTGAAGTTCGTACCACTGAGACATCCTGCCACCTTG | 2:191873833-191878261 | 111 | 60 | 97 |
| *STAT3* | Hs.463059 | NC_000017.10  NT_010783.15  NG_007370.1 | ENST00000264657  ENST00000404395  ENST00000389272 | GGTGTCACACAGATAAACTTGGTCTTCAGGTATGGGGCAGCGCTACCTGGGTCAGCTTCAGGATGCTCCTGGCTCTCTGGCCGACAATACTTTCCGAATGCCTCCTCCTTGGGAATGTCAGGATAGAGATAGACCAGTGGAGACACCAGGATATTGGT | 17:40469200-40474414 | 125 | 60 | 96 |
| *STAT4* | Hs.80642 | NC_000002.11  NG_012852.1  NT_005403.17 | ENST00000358470  ENST00000392320  ENST00000413064  ENST00000409995  ENST00000450994 | AGTTTTGAAGAAGAATCGTTGCCATGGTTTCATTGTTAGAAGCTGCCTCCCAGTCTTGATTTTCAATCCATTGGGCCAACAGATGCCGAATTTCCATGGGAAAGTTGTCAT | 2:192011434-192012862 | 81 | 60 | 94 |
| *STAT6* | Hs.524518 | NC_000012.11  NT_029419.12  NG_021272.1 | ENST00000300134 | ATAGACACATGTTCTATGTGGTCATGCAACTAAGGTGCCAGCTATACATTTAACATATCCTAGGTACATACACGTTCACACAGCTATACACGAAGAATCTCAGCCCTTGTACTTTTGCATAGTCTCATACACGTATCAGAAGCCTCCACC | 12:57489602-57489751 | 120 | 60 | 101 |
| *RORC* | Hs.256022 | NC_000001.10  NT_004487.19 | ENST00000356728  ENST00000318247  ENST00000392697 | GGAGGTGCTGGAAGATCTGCAGCCTTTCCACATGCTGGCTACACAGGCTCCGAAGCTTCCCCTTGGGTGGCAGCTTTGCCAGGATGCTTTGGCGATGAGTCTTGCAGAGATGATGATGAAAGGCCAGCTCCAGATTGTACTGCAGCTGTTCTA | 1:151780037-151783880 | 123 | 60 | 100 |
| *GATA3* | Hs.524134 | NC_000010.10  NG_015859.1  NT_008705.16 | ENST00000379328  ENST00000346208 | TGCAAAGGAGCTCACTGTGGTGTCTGTGTTCCAACCACTGAATCTGGACCCCATCTGTGAATAAGCCATTCTGACTCATATCCCCTATTTAACAGGGTC | 10:8116131-8116229 | 69 | 60 | 101 |
| *FOXP3* | Hs.247700 | NC_000023.10  NG_021311.1  NT_079573.4  NG_007392.1 | ENST00000376207  ENST00000557224  ENST00000518685  ENST00000376197  ENST00000376199  ENST00000455775 | GAAGGCAAACATGCGTGTGAACCAGTGGTAGATCTCATTGAGTGTCCGCTGCTTCTCTGGAGCCTCCAGGATGGCCCAGCGGATGAGCGTGGCGTAGGTGAAAGGGGGTCGCATGTTGTGGAACTTGAAGTAGTCCATGTTGTGGAGGAACTCTGGGAAT | X:49108152-49110385 | 129 | 60 | 102 |
| *NR4A2* | Hs.563344 | NC_000002.11  NG_011821.1  NT_005403.17 | ENST00000339562  ENST00000426264  ENST00000409572  ENST00000409108  ENST00000429376  ENST00000424077  ENST00000421709  ENST00000539077 | TAGTAAACCGACCCGGAGTGCGGCATCATCTCCTCAGACTGGGGGGGCAGGTGGCTGTGTTGCTGGTAGTTGTGCATCTGAATGTCTTCTACCTTAA | 2:157186334-157186430 | 67 | 60 | 99 |
| *RPS18* | Hs.627414 | NC_000006.11  NT_007592.15  NT_113891.2  NT_167245.1  NT_167247.1  NT_167248.1  NT_167249.1 | ENST00000454021  ENST00000486781  ENST00000484321  ENST00000211372  ENST00000477055  ENST00000476288  ENST00000439602  ENST00000474973  ENST00000457341  ENST00000494232  ENST00000434122 | GTGGAACGTGTGATCACCATTATGCAGAATCCACGCCAGTACAAGATCCCAGACTGGTTCTTGAACAGACAGAAGGATGTAAAGGATGGAAAATACA | 6:33243742-33243838 | 67 | 60 | 98 |
