# Supplementary material for: Parkinson’s disease patients have a complex phenotypic and functional Th1 bias: cross-sectional studies of CD4+ Th1/Th2/T17 and Treg in drug-naïve and drug-treated patients
Source: J Neuroinflammation. 2018 Jul 12;15:205. doi: 10.1186/s12974-018-1248-8 (PMC6044047; doi:10.1186/s12974-018-1248-8)
Supplement: Supplementary file 4 — Table S3. Complete blood count in HS and PD patients. Data are means ± SD unless otherwise indicated. (DOCX 45 kb) [file 12974_2018_1248_MOESM4_ESM.docx]

**Table S3.** **Complete blood count in** **HS and PD patients.** Data are means±SD unless otherwise indicated.

**A. Study #1**

|  | **units** | **range** | **HS** | **PD-dn** | **PD-dt** |  | **P** |  |
| --- | --- | --- | --- | --- | --- | --- | --- | --- |
|  |  |  |  |  |  | **HS vs PD-dn** | **HS vs PD-dt** | **PD-dn vs PD-dt** |
| **RBC** | 10^12^/L | 4.50-6.00 | 5.1±1.3 | 4.9±0.6 | 4.8±0.8 | 0.799 | 0.199 | 0.076 |
| **hemoglobin** | g/dL | 13.0-17.5 | 14.1±1.4 | 14.3±1.2 | 13.9±1.4 | 0.545 | 0.457 | 0.202 |
| **hematocrit** | % | 42.0-54.0 | 42.5±4.0 | 42.9±3.2 | 41.8±4.3 | 0.696 | 0.408 | 0.274 |
| **MCH** | pg | 27.0-32.0 | 30.4±2.4 | 29.9±1.7 | 30.1±2.2 | 0.595 | 0.999 | 0.652 |
| **MCHC** | g/dL | 32.0-36.0 | 33.5±2.4 | 33.6±1.9 | 33.4±1.6 | 0.692 | 0.858 | 0.578 |
| **Platelets** | 10^9^/L | 150-450 | 235.7±51.1 | 236.5±58.5 | 232.9±69.3 | 0.938 | 0.629 | 0.581 |
| **WBC** | 10^9^/L | 4.30-11.00 | 6.7±1.7 | 6.3±1.8 | 6.4±1.6 | 0.328 | 0.283 | 0.868 |
| ***lymphocytes*** | 10^9^/L | 1.50-5.50 | 2.1±0.6 | 1.7±0.4 | 1.8±0.5 | **0.004** | **0.007** | 0.318 |
|  | % | 10.0-45.0 | 30.1±8.4 | 26.1±8.5 | 28.9±7.2 | 0.057 | 0.433 | 0.129 |
| ***monocytes*** | 10^9^/L | 0.2-1.1 | 0.5±0.2 | 0.5±0.2 | 0.5±0.2 | 0.414 | 0.068 | 0.581 |
|  | % | 2.0-12.0 | 7.7±1.8 | 7.9±2.2 | 7.6±1.9 | 0.702 | 0.619 | 0.441 |
| ***neutrophils*** | 10^9^/L | 1.50-5.50 | 4.2±1.3 | 4.1±1.5 | 4.0±1.3 | 0.718 | 0.204 | 0.647 |
|  | % | 40.0-80.0 | 59.3±9.0 | 62.5±8.1 | 60.8±7.8 | 0.137 | 0.367 | 0.372 |
| ***eosinophils*** | 10^9^/L | 0.0-0.8 | 0.2±0.2 | 0.1±0.1 | 0.1±0.1 | 0.117 | 0.727 | 0.152 |
|  | % | 0.0-7.0 | 2.3±1.7 | 2.0±1.6 | 2.2±1.3 | 0.360 | 0.941 | 0.219 |
| ***basophils*** | 10^9^/L | 0.0-0.2 | 0.0±0.0 | 0.0±0.0 | 0.0±0.0 | 0.240 | 0.567 | 0.233 |
|  | % | 0.0-1.6 | 0.5±0.3 | 0.6±0.4 | 0.5±0.4 | 0.394 | 0.276 | 0.071 |

**Notes:** Abbreviations: RBC, red blood cells; MCH, mean corpuscular hemoglobin; MCHC, mean corpuscular hemoglobin concentration; WBC, white blood cells.

*(continues on next page)*

*(continuing from previous page)*

**B. Study #2**

|  | **units** | **range** | **HS** | **PD-dn** | **PD-dt** |  | **P** |  |
| --- | --- | --- | --- | --- | --- | --- | --- | --- |
|  |  |  |  |  |  | **HS vs PD-dn** | **HS vs PD-dt** | **PD-dn vs PD-dt** |
| **RBC** | 10^12^/L | 4.50-6.00 | 5.3±1.4 | 4.8±0.6 | 5.0±0.9 | 0.479 | 0.505 | 0.997 |
| **hemoglobin** | g/dL | 13.0-17.5 | 14.1±1.4 | 14.1±1.1 | 13.8±1.4 | 0.843 | 0.390 | 0.459 |
| **hematocrit** | % | 42.0-54.0 | 42.8±4.0 | 42.3±3.0 | 42.1±4.2 | 0.583 | 0.507 | 0.856 |
| **MCH** | pg | 27.0-32.0 | 30.1±1.9 | 29.8±1.7 | 29.3±2.5 | 0.560 | 0.213 | 0.417 |
| **MCHC** | g/dL | 32.0-36.0 | 33.4±2.8 | 33.6±1.9 | 33.2±2.3 | 0.883 | 0.637 | 0.231 |
| **Platelets** | 10^9^/L | 150-450 | 240.2±54.5 | 243.9±55.4 | 240.1±53.2 | 0.787 | 0.997 | 0.987 |
| **WBC** | 10^9^/L | 4.30-11.00 | 6.8±1.9 | 6.6±2.0 | 6.7±1.7 | 0.496 | 0.719 | 0.675 |
| ***lymphocytes*** | 10^9^/L | 1.50-5.50 | 2.2±0.6 | 1.8±0.5 | 2.0±0.5 | **0.004** | 0.299 | 0.092 |
|  | % | 10.0-45.0 | 28.9±8.1 | 26.5±7.8 | 29.2±6.6 | 0.241 | 0.854 | 0.130 |
| ***monocytes*** | 10^9^/L | 0.2-1.1 | 0.6±0.2 | 0.6±0.2 | 0.5±0.2 | 0.309 | 0.403 | 0.997 |
|  | % | 2.0-12.0 | 7.9±1.4 | 8.3±2.0 | 7.9±1.6 | 0.348 | 0.820 | 0.461 |
| ***neutrophils*** | 10^9^/L | 1.50-5.50 | 4.5±1.3 | 4.2±1.5 | 4.2±1.3 | 0.361 | 0.325 | 0.970 |
|  | % | 40.0-80.0 | 60.7±8.9 | 61.3±7.4 | 60.3±7.5 | 0.766 | 0.848 | 0.590 |
| ***eosinophils*** | 10^9^/L | 0.0-0.8 | 0.2±0.2 | 0.2±0.2 | 0.1±0.1 | 0.740 | 0.893 | 0.814 |
|  | % | 0.0-7.0 | 2.0±1.7 | 2.6±1.9 | 2.1±1.2 | 0.240 | 0.548 | 0.406 |
| ***basophils*** | 10^9^/L | 0.0-0.2 | 0.0±0.0 | 0.0±0.0 | 0.0±0.0 | 0.853 | 0.243 | 0.562 |
|  | % | 0.0-1.6 | 0.6±0.3 | 0.7±0.4 | 0.5±0.3 | 0.258 | 0.354 | 0.104 |

**Notes:** Abbreviations: RBC, red blood cells; MCH, mean corpuscular hemoglobin; MCHC, mean corpuscular hemoglobin concentration; WBC, white blood cells.
