# Supplementary material for: Parkinson’s disease patients have a complex phenotypic and functional Th1 bias: cross-sectional studies of CD4+ Th1/Th2/T17 and Treg in drug-naïve and drug-treated patients
Source: J Neuroinflammation. 2018 Jul 12;15:205. doi: 10.1186/s12974-018-1248-8 (PMC6044047; doi:10.1186/s12974-018-1248-8)
Supplement: Supplementary file 5 — Table S4. Lymphocyte count, comparison between HS and PD patients. Data are means ± SD unless otherwise indicated. Differences are indicated only when statistically significant, and are reported as the mean differences (with 95% confidence interval) between the means. (DOCX 48 kb) [file 12974_2018_1248_MOESM5_ESM.docx]

**Table S4.** **Lymphocyte count,** **comparison between HS and PD patients.** Data are means±SD unless otherwise indicated. Differences are indicated only when statistically significant, and are reported as the mean differences (with 95% confidence interval) between the means.

|  | **units** | **HS** | | | **PD** | | | **differences** | | |
| --- | --- | --- | --- | --- | --- | --- | --- | --- | --- | --- |
|  |  | **Kustrimovic**  **et al., 2016** | **Study #1** | **Study #2** | **Kustrimovic**  **et al., 2016** | **Study #1** | **Study #2** | **Kustrimovic**  **et al., 2016** | **Study #1** | **Study #2** |
| **lymphocytes** | 10^9^/L | 2.12±0.73 | 2.11±0.64 | 2.16±0.58 | 1.76±0.48 | 1.76±0.46 | 1.87±0.48 | -0.36  (-0.09 to -0.63) | -0.36  (-0.16 to -0.55) | -0.29  (-0.07 to -0.50) |
|  | % of WBC | 30.8±7.4 | 30.1±8.4 | 28.9±8.1 | 27.3±6.8 | 28.0±7.7 | 28.0±7.2 | -3.5  (-0.2 to -6.7) |  |  |
| **CD3+** | 10^6^/L | 1515.0±651.7 |  |  | 1241.0±366.2 |  |  |  |  |  |
|  | % of lymph | 70.8±9.4 |  |  | 69.6±8.7 |  |  |  |  |  |
| **CD4+** | 10^6^/L | 1012.0±439.1 | 1004.0±392.8 | 1097.0±347.1 | 797.4±263.3 | 759.6±264.7 | 827.7±277.0 | -214.5  (-59.2 to -369.8) | -244.0  (-129.2 to -358.8) | -268.9  (-141.1 to -396.6) |
|  | % of CD3+ | 67.3±10.8 | 47.0±9.0 | 50.6±8.1 | 64.7±10.8 | 43.0±9.2 | 44.1±8.4 |  | -4.0  (-0.7 to -7.3) | -6.5  (-2.9 to -10.0) |
| **CD8+** | 10^6^/L | 334.0±162.8 |  |  | 274.9±151.5 |  |  |  |  |  |
|  | % of CD3+ | 23.4±9.3 |  |  | 22.0±9.7 |  |  |  |  |  |
| **CD4+/CD8+** | ratio | 3.8±2.9 |  |  | 4.0±3.4 |  |  |  |  |  |
| **T naive** | 10^6^/L | 396.4±263.0 |  |  | 279.2±141.4 |  |  | -117.2  (-28.0 to -206.4) |  |  |
|  | % of CD4+ | 37.2±7.3 |  |  | 34.2±10.2 |  |  |  |  |  |
| **T_CM_** | 10^6^/L | 243.2±93.2 |  |  | 215.1±85.8 |  |  |  |  |  |
|  | % of CD4+ | 24.8±5.0 |  |  | 27.5±6.9 |  |  |  |  |  |
| **T_EM_** | 10^6^/L | 274.3±100.3 |  |  | 249.1±91.8 |  |  |  |  |  |
|  | % of CD4+ | 27.9±5.5 |  |  | 31.2±7.8 |  |  | 3.3  (0.0 to 6.6) |  |  |
| **Th1** | 10^6^/L |  | 149.1±90.1 |  |  | 140.5±95.4 |  |  |  |  |
|  | % of CD4+ |  | 14.6±6.1 |  |  | 18.1±9.0 |  |  | 3.5  (0.1 to 6.8) |  |
| **Th2** | 10^6^/L |  | 78.7±63.7 |  |  | 54.9±35.2 |  |  | -23.7  (-4.3 to -43.1) |  |
|  | % of CD4+ |  | 7.8±4.2 |  |  | 7.3±3.7 |  |  |  |  |
| **Th1/Th2** | ratio |  | 2.5±1.7 |  |  | 3.1±2.3 |  |  |  |  |
| **Th17** | 10^6^/L |  | 89.3±48.5 |  |  | 58.2±27.4 |  |  | -31.1  (-16.2 to -46.0) |  |
|  | % of CD4+ |  | 9.3±4.1 |  |  | 7.9±3.2 |  |  |  |  |
| **Th1/Th17** | 10^6^/L |  | 112.9±67.0 |  |  | 79.1±46.5 |  |  | -33.8  (-11.5 to -56.2) |  |
|  | % of CD4+ |  | 11.7±5.7 |  |  | 10.8±4.9 |  |  |  |  |
| **Treg** | 10^6^/L |  |  | 98.4±35.6 |  |  | 67.9±20.5 |  |  | -30.4  (-19.3 to -41.6) |
|  | % of CD4+ |  |  | 9.2±2.6 |  |  | 8.6±2.3 |  |  |  |
| **nTreg** | 10^6^/L |  |  | 21.8±10.2 |  |  | 22.7±7.3 |  |  | -5.7  (-2.4 to -9.0) |
|  | % of Treg |  |  | 22.4±6.3 |  |  | 33.7±6.2 |  |  |  |
| **aTreg** | 10^6^/L |  |  | 33.7±15.3 |  |  | 16.2±6.3 |  |  | -11.0  (-6.5 to -15.5) |
|  | % of Treg |  |  | 33.9±6.9 |  |  | 23.8±6.0 |  |  |  |
